# Gut diversity and the resistome as biomarkers of febrile neutropenia outcome in paediatric oncology patients undergoing hematopoietic stem cell transplantation

**DOI:** 10.1038/s41598-024-56242-8

**Published:** 2024-03-06

**Authors:** Sara Sardzikova, Kristina Andrijkova, Peter Svec, Gabor Beke, Lubos Klucar, Gabriel Minarik, Viktor Bielik, Alexandra Kolenova, Katarina Soltys

**Affiliations:** 1 Department of Microbiology and Virology, Faculty of Natural Sciences, Comenius University in Bratislava, Bratislava, Slovakia; 2 Department of Paediatric Haematology and Oncology, Children’s Haematology and Oncology Clinic and Faculty of Medicine, Comenius University in Bratislava, Bratislava, Slovakia; 3 Institute of Molecular Biology, Slovak Academy of Sciences, Bratislava, Slovakia; 4 Medirex Group Academy N.P.O., Nitra, Slovakia; 5 Department of Biological and Medical Science, Faculty of Physical Education and Sport, Comenius University in Bratislava, Bratislava, Slovakia

**Keywords:** Alpha-diversity, Gut microbiome, Resistome, Multidrug-resistant bacteria, Paediatric oncology, Hematopoietic stem cell transplantation, Cancer, Computational biology and bioinformatics, Microbiology, Molecular biology

## Abstract

The gut microbiota of paediatric oncology patients undergoing a conditioning regimen before hematopoietic stem cell transplantation is recently considered to play role in febrile neutropenia. Disruption of commensal microbiota and evolution of opportune pathogens community carrying a plethora of antibiotic-resistance genes play crucial role. However, the impact, predictive role and association of patient´s gut resistome in the course of the therapy is still to be elucidated. We analysed gut microbiota composition and resistome of 18 paediatric oncology patients undergoing hematopoietic stem cell transplantation, including 12 patients developing febrile neutropenia, hospitalized at The Bone Marrow Transplantation Unit of the National Institute of Children´s disease in Slovak Republic and healthy individuals (n = 14). Gut microbiome of stool samples obtained in 3 time points, before hematopoietic stem cell transplantation (n = 16), one week after hematopoietic stem cell transplantation (n = 16) and four weeks after hematopoietic stem cell transplantation (n = 14) was investigated using shotgun metagenome sequencing and bioinformatical analysis. We identified significant decrease in alpha-diversity and nine antibiotic-resistance genes *msr(C), dfrG, erm(T), VanHAX, erm(B), aac(6)-aph(2), aph(3)-III, ant(6)-Ia* and *aac(6)-Ii*, one week after hematopoietic stem cell transplantation associated with febrile neutropenia. Multidrug-resistant opportune pathogens of ESKAPE, *Enterococcus faecium*, *Staphylococcus aureus*, *Klebsiella pneumoniae* and* Escherichia coli* found in the gut carried the significant subset of patient’s resistome. Over 50% of patients treated with trimethoprim/sulfamethoxazole, piperacillin/tazobactam and amikacin carried antibiotic-resistance genes to applied treatment. The alpha diversity and the resistome of gut microbiota one week after hematopoietic stem cell transplantation is relevant predictor of febrile neutropenia outcome after hematopoietic stem cell transplantation. Furthermore, the interindividual diversity of multi-drug resistant opportunistic pathogens with variable portfolios of antibiotic-resistance genes indicates necessity of preventive, personalized approach.

Antibiotic resistance is a worldwide problem, although geographic and institution-level differences are observed (https://atlas-surveillance.com/#/heatmap/resistance) (accessed on 4 March 2021). This phenomenon also affects paediatric patients receiving allogeneic hematopoietic stem cell transplantation (HSCT) who become at risk of receiving an inadequate initial empirical therapy for febrile neutropenia, with an increased likelihood of complicated clinical course^1^. Patients undergoing HSCT are known to suffer from severe gut microbiota dysbiosis associated with reverse outcomes, including febrile neutropenia, after HSCT. The recipients of HSCT belong to a group representing patients awaiting novel microbiota therapy^2^. For most adult patients, loss of diversity and increased abundance of *Enterococcus* spp. is a common feature, while in acute leukaemia, there is also an increase of *Lactobacillus*^3^. Gut microbiota is intensively studied in adults but lacks paediatric patients' cohorts undergoing HSCT for its association with adverse transplantation outcomes, including bacteraemia, viremia, or fungemia ^4^. Antibiotic resistance can lead to clinical response failure ^5^ and influence cancer treatment through drug metabolism ^6^. Gut microbiota is considered a rich source of antibiotic-resistant bacteria that, in healthcare settings, accumulate and spread during prolonged exposure to antibiotics. Thanks to their genes for antimicrobial resistance that enable the survival of commensal and opportune pathogens during a patient's treatment, they can influence the clinical outcomes after HSCT through molecular interactions or gut barrier. Despite advances in molecular diagnostics, identifying meaningful biomarkers, either predictive or prognostic, is rare. The overall decrease in diversity of gut microbiota and enrichment with *Enterococcaceae* and *Streptococcaceae* has been considered a predictor of diarrhoea^7^, *Faecalibacterium* at the baseline of an infection classifier^8^. At the same time, the higher abundance of *Proteobacteria* was associated with febrile neutropenia (FN)^7^. Most infections result from antibiotic-resistant bacterial strains^4,9^, and the status and the recovery of the immune system after HSCT play a crucial role in the incidence and severity of infection. Two clinical trials stress the effects of piperacillin-tazobactam on gut microbiota depletion (ClinicalTrials.gov Identifiers: NCT03078010/NCT02641236) during FN. In contrast, using fluoroquinolones during neutropenia has been associated with findings^10^ stating that *Streptococcus*, *Staphylococcus*, *Klebsiella,* and *Escherichia* are the most common drivers of bacteraemia. The resistome of paediatric patients during HSCT is highly variable and dynamic^11^.

Febrile neutropenia is a clinical syndrome characterizing patients with fever and neutrophil count = granulocyte count < 500/mm3 or < 1000/mm3 with a decrease expected to fall below 500/mm3. Definitions of fever vary, but by default, fever can be considered an ascent of body temperature above 38 °C lasting at least 1 h, or two independent outputs of body temperature above 38 °C within 12 h, or a single outlet of fever above 38.5 °C. Rarely, infection during neutropenia may present itself without fever^12^. Febrile neutropenia, as a reverse outcome of live-saving hematopoietic stem cell transplantation, in the majority of cases, is a consequence of bloodstream infection caused by microorganisms, including gram-positive bacteria (*E. faecium, E. faecalis, S. aureus*) and gram-negative rods and yeasts^13^. Furthermore, it is the leading cause of morbidity and mortality in these patients. Gut microbiota is recently considered to be associated with hematopoietic stem cell transplantation. So far, the gut dysbiosis profile and resistome of 39 paediatric oncology patients have been described^14^. However, the shifts in its composition or diversity before and after HSCT were not. On the contrary, the gut microbiota dysbiosis before/after HSCT and its resistome were characterized, but only in 8 patients with a focus on graft-versus-host disease (GvHD) as the reverse outcome of HSCT^15^, and currently, the gut microbiota diversity before HSCT has been shown as a biomarker of GvHD after HSCT^16^. Regarding the length of fever during febrile neutropenia, it has been associated with the severity of gut microbiota dysbiosis^17^, and according to Schwabkey^18^ and colleagues (2022), it is linked to diet, metabolites, and colonic mucus also through *Akkermansia muciniphila*. We have investigated the effect of HSCT treatment on gut microbiota composition in children with acute lymphoblastic leukaemia and the impact of physical exercise on its composition and found that CRP correlated positively with the pathogenic bacteria *Enterococcus* spp. and negatively with beneficial bacteria *Butyriccocus* spp. or *Akkermansia* spp^19^.

Although there is good practice for antibiotic-resistant bacteria (ARB) screening from different body sites, routine screening has been established mainly through culture-dependent methods. Screening patients for infectious diseases is crucial before and after transplantation to prevent the spread of highly virulent microorganisms. Comprehensive screening procedures should assess potential threats such as methicillin-resistant *S. aureus*, vancomycin-resistant enterococci, and multidrug-resistant gram-negative bacteria^20^. Gut microbiota is often neglected as a potential source of ARB and is investigated using rectal swabs cultivation analysis. However, this approach only allows incomplete characterization of the whole gut resistome that is dynamic and specific for an individual. Since the horizontal transfer of genes to pathogens or opportune pathogens is associated with the uprise of multidrug-resistant species^21,22^, regular screening of antimicrobial resistance (AMR) of gut microbiota and personalized treatment is highly desirable.

With a worldwide increase in antibiotic resistance^23^, also paediatric patients undergoing HSCT^24^ are at high risk of receiving inappropriate standard empirical therapy for FN, with an increased likelihood of a complicated clinical course^1,25–30^.

It has been estimated that bloodstream infections due to gram-negative bacteria resistant to more than three antibiotics were significantly associated with intensive care unit admission and death^13^.

Knowledge of the gut resistome profile of paediatric oncology patients and the epidemiology of antibiotic-resistant bacteria is urgently needed to determine the best personalized therapeutical approach and patient management^13^. Our study aimed to use metagenomic sequencing, which is considered a susceptible and reliable method for AMR screening^31^, to perform bacteriome profiling and resistome characterization of the gut microbiota of paediatric oncology patients undergoing HSCT in three time-points, before transplantation, one week after transplantation and one month after transplantation. We aimed to investigate the gut diversity, resistome dynamics during hospitalization and performed functional prediction of antibiotic resistance through gene categorization and clustering. Furthermore, we have identified bacterial species carrying ARGs and investigated the relevance of antimicrobial therapy and the potential involvement of resistant bacteria in chemotherapy through drug metabolism. According to our knowledge, this is the first study of such size to identify the full potential of gut microbiota focused on resistome of paediatric patients undergoing HSCT with a portfolio of resistant bacterial species investigating their potential involvement in HSCT therapy.

## Methods

### Subjects

61 faecal samples were obtained from randomly assigned 18 paediatric oncology patients at the Department of Paediatric Haematology and Oncology-Bone Marrow Transplant Unit (Faculty of Medicine at Comenius University and National Institute of Child Diseases) from 2019 to 2021. The samples were collected seven days before HSCT (d-7), one week (d + 7), and one month (d + 28) after HSCT. Only two samples were available from four patients; two provided samples before and one week after HSCT (d-7 and d + 7), and two of them one week and one month after HSCT (d + 7 and d + 28). A single sample (d-7) was available from two patients. The cohort of patients included 11 males and seven females from one to 15 years of age (10.5 ± 5.7 years). Eleven patients were diagnosed with acute lymphoblastic leukaemia, three with acute myeloid leukaemia, two with myelodysplastic syndrome, one with anaplastic large cell lymphoma, and one with X-linked lymphoproliferative disease. Twelve patients developed FN, and nine patients were diagnosed with GvHD, providing six of them had been diagnosed with both GvHD and FN (Supplementary Table 1). Samples from fourteen healthy children (six males, eight females) aged four to 18 (8.5 ± 4.4 years) were collected in 2021.

Patients in our study experienced febrile neutropenia within d + 1 to d + 15 after HSCT. The duration of febrile neutropenia varied from 1 to 25 days. All fecal samples were collected from 0 to 7 days after FN onset and were always obtained as soon as they were available. Only from one patient developing FN 15 days post-HSCT, lasting for two days, was a stool sample obtained after 14 days; notwithstanding this fact, this patient was included in the study, matching the microbiome composition with patients from the appropriate cohort.

Randomly assigned healthy individuals had not been diagnosed with any acute or chronic disease and had not received any antimicrobial treatment for one month before sampling. The legal representatives of all participants signed informed consent. All analysis carried out within the study were blinded. For the study purposes, patients were included provided at least two biological specimens were obtained within the period of HSCT therapy.

### Febrile neutropenia treatment with antimicrobials

The treatment regimens involved a combination of broad-spectrum antibiotics. The choice of antibiotics could reflect the underlying conditions, severity of infection or antibiotic susceptibility patterns. Standard antibiotic therapy in febrile neutropenia included Piperacillin-Tazobactam + Amikacin. Gram-positive bacteria coverage was added if central venous line (CVL) infection was suspected, skin infection was present, or fever last more than three days. For this purpose, Piperacillin, Tazobactam, and Amikacin were used. Meropenem was used if Piperacillin or Tazobactam-resistant bacteria were detected or febrile neutropenia last or worsened on the standard antibiotic combination for a couple of days. Linezolid was preferred over teicoplanin if soft tissue or lung infection with Gram-positive bacteria was present or possible. Trimethoprim-sulfamethoxazole before HSCT was used two or three days per week as prophylaxis of *Pneumocystis jirovecii* infection. It was paused during aplasia and re-introduced after stable engraftment on new hemopoiesis. The combination of applied antibiotic treatment varied across patients reflecting the cultivation-based microbiological findings (Supplementary Table 2.).

### Sample collection, DNA isolation and high-throughput sequencing

Stool samples were collected and stored at − 80 °C until further processing. The extraction of DNA was provided by Zymobiomics DNA/RNA isolation kit (ZymoResearch, Irvine, CA) according to the manufacturer's protocol. Extracted DNA was quantified with Qubit dsDNA High Range Assay (Thermo Fisher Scientific, Waltham, MA, USA) using Qubit 4.0 Fluorometer (Invitrogen, Carlsbad, CA, USA). DNA was eluted and stored at − 20 °C. DNA libraries were prepared using Nextera kit (Illumina, San Diego, CA, USA) according to the manufacturer’s instructions. Fragmenation was followed with indexing PCR and the amplified libraries were purified using Agencourt AMPure beads (Beckman Coulter, Indiana, USA). The quality and the quantity of final libraries were established by Qubit 4.0 Fluorometer and chip electrophoresis using Agilent Bioanalyser 2100 system (Agilent Technologies, Santa Clara, CA, USA). Samples were pooled in equimolar ratio and sequenced on Illumina NextSeq (Illumina, San Diego, CA, USA) platform (2 × 150 bp). All steps were performed according to the manufacturer's tutorials.

### Bioinformatic analysis

Raw reads were pre-processed (i.e., quality control and trimming) using an internal Galaxy web instance^32^ (https://usegalaxy.org/). Read quality reports were generated using the FastQC tool^33^ (https://github.com/s-andrews/FastQC). Trimming was performed with Trimmomatic^34^ (https://github.com/usadellab/Trimmomatic)—Galaxy Version 0.38.1, parameters: CROP:296, HEADCROP:17, MINLEN:50. For metagenomic assembly metaSPAdes^35^ (https://github.com/ablab/spades) was used, with default parameters. For taxonomical classification of trimmed reads and metaSPAdes scaffolds, Kraken 2 [https://github.com/DerrickWood/kraken2] with a custom-built database, which contains archaea, bacteria, plasmid, viral, human, fungi, protozoa, and UniVec_core Kraken 2 sequence libraries (https://benlangmead.github.io/aws-indexes/k2, retrieved on March 8, 2023) was used (Supplementary Table 3). The database was built according to the Kraken 2 protocol^36^. Kraken2 output files were converted to BIOM format using the kraken-biom tool (https://github.com/smdabdoub/kraken-biom)^37^. Following the phyloseq (https://joey711.github.io/phyloseq/) tutorials, we imported the converted kraken2 BIOM tables into R environment (version 4.2.2)^38^ (https://www.r-project.org/) using the *phyloseq* library (version 1.42.0)^39^. Alpha and beta diversities were calculated and plotted with the *phyloseq* library (all default parameters). Beta diversities calculations were carried out with PERMANOVA, Bray–Curtis distance matrix in R and *vegan* package (https://github.com/vegandevs/vegan) version 2.6–4 libraries, significance *p* ≤ 0.05 was considered.

### Identification of drug resistance genes and antimicrobial substance resistance prediction

Sequence reads assembled as contigs were used as input in the ResFinder (https://cge.food.dtu.dk/services/ResFinder/) for the resistance gene identification^40–42^. All acquired antimicrobial resistance genes were identified provided they overlapped with the reference sequence for 100% and classified based on a minimum 98% identity with the reference gene. The presence/absence of a gene expressed as 1 / 0 was used for further statistical analysis. Resistance genes were associated with antimicrobial substance resistance through predicted phenotype, categorization into antimicrobial classes, and clustering in the ResFinder database (2022-05-24). The ABRIcate tool (https://github.com/tseemann/abricate) for the virulence gene identification using vfdb (2019-04-19) database was used.

### Identification and taxonomic classification of resistant bacteria

The individual contigs with detected resistance genes were analysed through BLAST^43^ (optimized for highly similar sequences (megablast)) using a nucleotide collection database, and the sequence with the highest identity (min. 99%) and coverage score was considered the closest relative. The position of the resistance gene within the bacterial genome (chromosome/plasmid) was recorded.

### Bacteria-cancer drug interaction

ResFinder-identified resistant bacteria were searched in the databases http://pharmacomicrobiomics.com/ and http://www.aiddlab.com/MASI/. All information on bacteria-drug interactions came exclusively from databases. Only bacteria associated with anticancer therapy taken by studied patients were selected. Patients' data were screened for taken antibiotics. The effectiveness of the therapy taken, and post-transplant FN was associated with the resistant bacteria of the gut.

### Statistical analysis

The data were analysed by SPSS (IBM Corp. Released 2012. IBM SPSS Statistics for Windows, Version 21.0. Armonk, NY: IBM Corp.) as well as the LefSe tool^44^. Shapiro–Wilk test was used for the normality testing. Based on this result, Kruskal–Wallis or Mann–Whitney test, respectively, were used as non-parametric tests, while the t-test or ANOVA were used as parametric tests. The tests were provided with established α-level. Correlation analysis was based on the Spearman rank correlation coefficient. Interaction networks were visualized with Gephi (Gephi version 0.10.1, www.gephi.org). The Gephi network was made using ‘Force Atlas’ layout and ranking used ‘Average Path Length’ algorithm with ‘Betweeness Centrality’ parameter. ‘Modularity class' algorithm was used for the community detection and the values were used to colorize communities. GraphPad (GraphPad Prism version 8.0.0 for Windows, GraphPad Software, San Diego, California USA, www.graphpad.com) was used for data visualization. Moreover, the Krona pie chart^45^ was used for microbial composition visualization. The clustVis tool^46^ was used for Principal Component Analysis and heatmap visualization using average clustering for antimicrobial genes based on correlation distance and for patient´s samples Ward clustering based on Manhattan clustering distance. Adobe Illustrator 2020 enabled us picture creation and editing.

### Ethics approval

The study adhered to the ethical principles outlined in the Declaration of Helsinki for experiments involving human beings and its later amendments, and all steps of this study were approved by the Ethics Committee of the National Institute of Child Diseases (NICD-25/4/18). The legal representatives of all participants provided and signed written informed consent.

## Results

### Change in alpha-diversity of gut microbiota one week after HSCT is associated with febrile neutropenia

**Figure 1:**
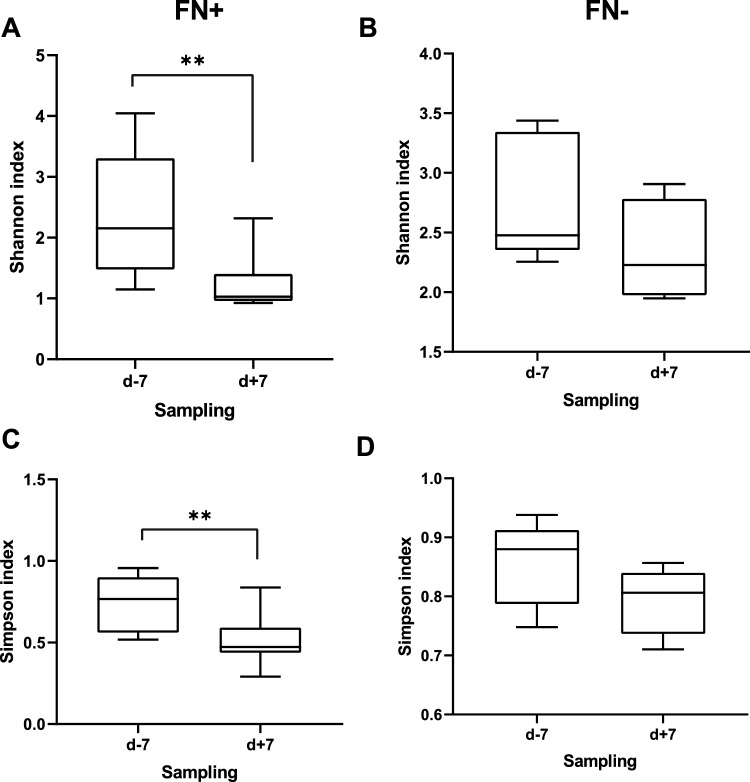
Alpha diversity of gut microbiome of patients without febrile neutropenia (FN-) or developing febrile neutropenia (FN+) before (d-7) and after (d + 7) HSCT represented by Shannon index (**A**, **B**) and Simpson index (**C**, **D**). The p values were computed using Mann–Whitney test for parametric data and Wilcoxon-rank test for nonparametric data with significance p ≤ 0.05 applied. Significant decrease in alpha diversity was detected in gut microbiome of patients developing febrile neutropenia after HSCT.

Shannon and Simpson indices were used for alpha-diversity determination. The diversity of the healthy gut microbiome (median ± interquartile range) defined by Shannon index (4.27 ± 3.83) and Simpson index (0.96 ± 0.93) represented the benchmark for the evaluation of the deviated diversity observed in gut of patients. Significantly lower alpha diversity defined by both indices characterized both groups of patients, either with or without FN developed after HSCT, during the monitored period compared to healthy individuals (*p* ≤ 0.01). Although there was no significant difference between the two cohorts of patients within the same period of analysis, overall species richness (Simpson index; d-7/d + 7/d + 28) of the gut microbiome was less favourable for patients with FN (0.77 ± 0.56/0.47 ± 0.43/0.48 ± 0.29) compared to the other group (0.88 ± 0.78/0.80 ± 0.73/0.63 ± 0.41) pinpointing an important role of the alpha diversity in association with posttransplant complication. Furthermore, one week after HSCT, patients developing FN exhibited a significant decrease in alpha diversity app. by 60% compared to the timepoint before HSCT (Simpson; *p* = 0.016), whereas no significant change was observed in patients without FN (Fig. 1). The analysis of Shannon index provided similar results (*p* = 0.002) proving remarkable gut microbiota depletion after HSCT preceding FN (2.15 ± 1.47/1.03 ± 0.95/1.05 ± 0.72) compared to the group without fever development (2.47 ± 2.35/2.22 ± 1.97/1.57 ± 1.22). We haven´t detected any specific antimicrobial or antimicrobials combination that would be associated with selective depletion of gut microbiota after HSCT in patients developing fever. Although, consequently individually set therapy with antimicrobials was applied to FN treatment. Significantly different beta diversity discriminated healthy individuals from patients either developing or not FN before HSCT (*p* ≤ 0.001) as well as within one week after HSCT (*p* ≤ 0.001), while no difference was observed between the two cohorts of patients before HSCT (*p* ≤ 0.28) or after HSCT (*p* ≤ 0.25). The intragroup diversity did not change after treatment in any of involved groups of patients (with FN *p* = 0.4; without FN *p* = 0.5).

### Increase in Bacillota phylum in gut microbiota of patients receiving HSCT

**Figure 2:**
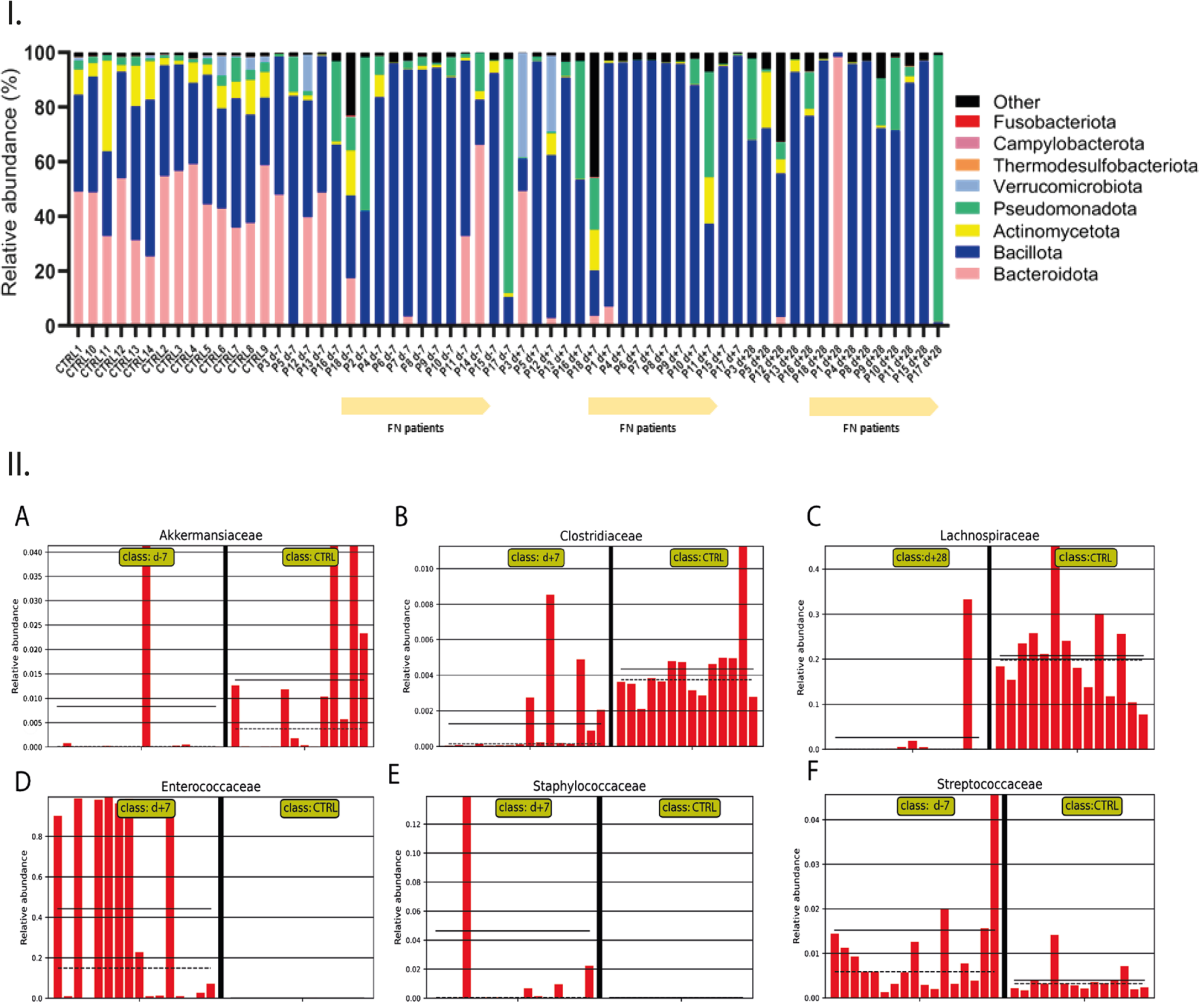
Gut microbiome composition at phylum level (I.) calculated as relative abundance visualized as bargraphs. All involved participants including healthy individuals and patients at all time-points of sampling were included. II. – relative abundance of selected bacterial families of gut microbiota of patients before (d-7), one week after (d + 7) and four weeks after (d + 28) compared to healthy individuals (CTRL). Beneficial commensal bacteria (**A**—*Akkermansiaceae*, **B**—*Clostridiaceae*, **C**—*Lachnospiraceae*) and opportunistic pathogens (**D**—*Enterococcaceae*, **E**—*Staphylococcaceae*, **F**—*Streptococcacea*e) visualized as bar charts. Significant decrease (p ≤ 0.05) in relative abundance of *Bacteroidota, Akkermansiaceae, Clostridiacea, Lachnospiracea* and increase of opportunistic pathogens was observed in gut microbiome of paediatric oncology patients.

The gut microbiota composition was assessed at crucial timepoints—before HSCT (d-7), one week after HSCT (d + 7), and one month after HSCT (d + 28). This period, marked by conditioning regimen, inclusion of GvHD, and antimicrobial prophylaxis, possessed changes to the microbiome's stability. *Bacillota* dominated, representing 64% before HSCT and escalating to 76% one week after. *Pseudomonadota* decreased to 7% from 13%, while *Bacteroidota* decreased to 4% from 15%. A newly emerged phylum *Verrucomicrobiota* accounted for 4%, and *Actinobacteria* showed almost no change with an abundance of 2.7%. One month after HSCT, *Bacillota* decreased to 70%, *Pseudomonadota* maintained 13%, while *Bacteroidota* increased to 7%. *Actinomycetota* stayed with approximately the exact count (2.4%). The abundances of the other nine phyla remained below 1% (Fig. 2).

The bacterial profile of the patient’s gut microbiome either before or after HSCT revealed a significant decrease (*p* ≤ 0.05) in the relative abundance of *Bacteroidota, Actinomycetota*, and *Thermodesulfobacterota* and an increase in *Bacillota*. Furthermore, a disbalance of *Fusobacteriota* and a decrease of *Campylobacteriota* were detected in a subgroup of patients mainly observed before and one week after HSCT (Supplementary Table 4).

### Impact of HSCT on gut microbiota composition one week after HSCT

The analysis of the family level (Supplementary Table 5) provided more specific insight into the changes in the patient’s gut microbiota.

Before HSCT, *Lachnospiraceae* (24%) and *Enterococcaceae* (23%) dominated, with other families like *Enterobacteriaceae* (12%), *Bacteroidaceae* (11%), and *Oscillospiraceae* (7%) present. One week post-HSCT, *Enterococcaceae* surged to 41%, while *Lachnospiraceae* dropped to 12%. *Streptococcaceae*, *Lactobacillaceae*, and *Staphylococcaceae* appeared at 5%, and *Akkermansiaceae* and *Coprobacillaceae* at 3%. *Enterobacteriaceae* dropped to 3%, while *Pseudomonadaceae* increased to 2%. After one month, *Enterococcaceae* still dominated (41%), followed by *Enterobacteriaceae* (11%), and increased *Staphylococaceae* and *Streptococcaceae* (9%). *Bacteroidaceae*, *Carnobacteriaceae*, and *Lachnospiraceae* were also identified (2%).

Among all identified OTUs, *E. faecium* emerges as the dominant species in the gut microbiota before HSCT, comprising 15% of the total microbial abundance. Other commensal bacteria detected with higher abundances included *[Ruminococcus] gnavus* (6.7%), *Enterocloster bolteae* (5.4%), *Phocaeicola vulgatus* (4.5%), *[Clostridium] innocuum* (3.1%), *Flavonifractor plautii* (2.9%), *Roseburia intestinalis* (2%), *Parabacteroides distasonis* (1.9%), *Blautia* spp. NBRC 113,351 (2%), *Faecalibacterium prausnitzii* (1.9%), *Enterocloster clostridioformis* (1.1%), and *Sellimonas intestinalis* (1.1%). Additionally, *Citrobacter farmers* (2.1%) belonging to the opportunistic pathogens was detected alongside *E. faecium* (Fig. 2).

The prevalence of *E. faecium* increased one week after HSCT (32.4%) and represented the most abundant species in patients. There was also an increase observed in *Akkermansia muciniphila* (3.9%), which is considered to be the protector of the gut barrier^47^, *Latilactobacillus sakei* (3.1%), and *Blautia* spp. NBRC 113,351 (2.4%). However, an increased abundance of *Staphylococcus haemolyticus* (3.6%) could be a potential concern in these samples. A subtle decrease was detected in commensal bacteria *E. bolteae* (3.9%), *[Ruminococcus] gnavus* (1.5%), *P. distasonis* (1.6%), *F. plautii* (1.7%), *[Clostridium] innocuum* (1.6%).

**Figure 3:**
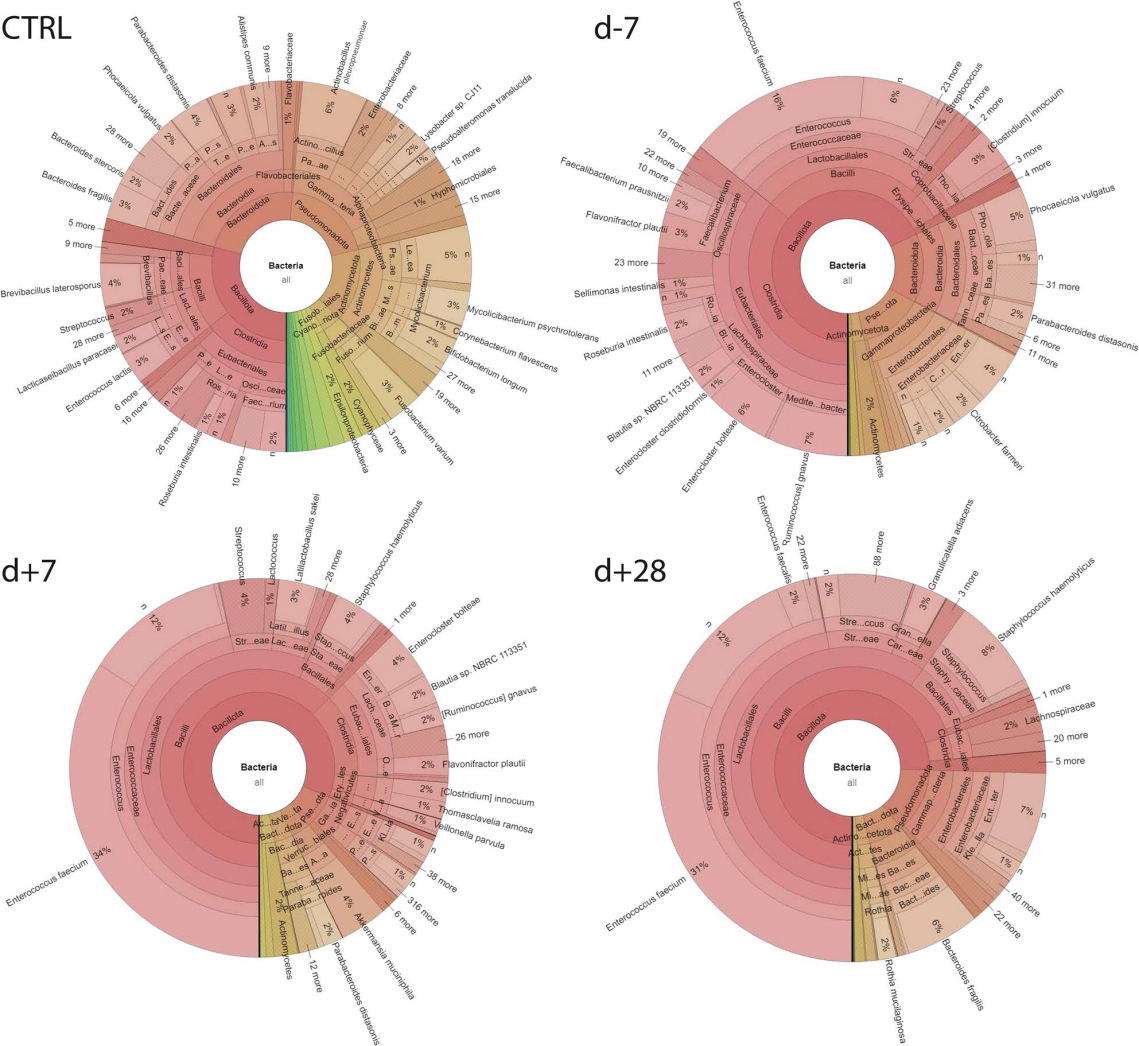
The gut bacteriome of the patients undergoing HSCT and healthy individuals visualized using a Krona pie chart. Each chart represents gut composition calculated as average for each bacterial genus detected within the group. CTRL—control group; d-7—before HSCT; d + 7—one week after HSCT; d + 28—one month after HSCT.

One month after treatment, *E. faecium* remained a dominant species in gut microbiota, with an abundance of 30%. Additionally, there was a pronounced increase in the abundance of *Enterococcus faecalis* by 2.2%, and a similar increase of 7.1% was observed for *S. haemolyticus*. All the mentioned bacterial species, together with *Granulicatella adiacens* (2.6%), *Rothia mucilaginosa* (1.2%), and *Bacteroides fragilis* (5.8%) were detected with an abundance higher than 1% (Fig. 3).

### Gut microbiome of healthy children

The gut microbiota of the healthy group was characterized by the most abundant phylum *Bacteroidota* (45%) and phylum *Bacillota* (40%). *Actinomycota* was detected with an abundance of 10%, *Pseudomonadota* was identified with an abundance of 3%, and *Verrucomicrobiota* with 1%.

While patients showed a significant increase in families associated with potential opportunistic pathogens, the gut microbiome of healthy controls was primarily dominated by *Bacteroidaceae* and *Lachnospiraceae*, accounting for 20% of the total abundance, followed by *Oscillospiraceae* (15%), *Prevotellaceae* (11%) and *Bifidobacteriaceae* (7%).

The healthy control group shows a diverse microbial composition, with various bacterial species including *Brevibacillus laterosporus* (3.7%), *P. distasonis* (3.5%), *B. fragilis* (3.1%), *Enterococcus lactis* (2.7%), *P. vulgatus* (2.2%), *Alistipes communis* (1.7%), *Lacticaseibacillus paracasei* (1.6%), *Bacteroides stercoris* (1.5%), *Acidobacterium capsulatum* (1.3%), and *Ilyobacter polytropus* (1.1%) were the most abundant bacteria in the healthy group. *Fusobacterium varium* (3%) and *Corynebacterium flavescens* (1.1%), correlated to infectious potential, were also healthy children's most abundant gut microbiota members (Figs. 2, 3).

### Low-abundance opportune pathogens in microbiome of cancer patients as potential source of infection

However, not only dominant bacteria but also low-abundance bacterial species influence and reflect the gut dysbiosis level through exhibiting higher resistance to commonly used broad-spectrum antimicrobials, posing a higher risk for immunocompromised patients, as they may possess the ability to evade the antimicrobial prophylaxis. We have focused on specific bacterial taxa that could carry the resistance genes. We have identified that the *Bacilli* class included opportunistic pathogens *E. faecalis* with an average abundance of 0.05% across samples one week after HSCT, while 0.55% in samples before HSCT. *Enterococcus hirae* was detected in all three time points with an abundance < 1% (0.04%/0.05%/0.05%). The potential pathobionts of the *Streptococcus* genus included *Streptococcus agalactiae* (0.002%/0.003%/0.01%), *Streptococcus gallolyticus* (0.0004%/0.0005%/0.002%), *Streptococcus infantis* (0.01%/0.19%/0.54%), *Streptococcus pneumoniae* (0.02%/0.06%/0.1%) and *Streptococcus suis* (0.005%/0.01%/0.03%). The genus *Staphylococcus*, another member of class *Bacilli*, was represented by three species: *Staphylococcus aureus* (0.3%/0.04%/0.07%), *Staphylococcus epidermidis* (0.02%/0.16%/0.07%) and *Staphylococcus hominis* (0.002%/0.02%/0.1%). Furthermore, we have detected *Clostridioides difficile* (0.09%/0.05%/0.02%), the well-known opportune pathogen of class *Clostridia*, and four members of the *Gammaproteobacteria* class (phylum *Pseudomonadota*) involving clinically relevant *E. coli* (0.25%/0.07%/0.07%), *Enterobacter hormaechei* (0.24%/0.02%/0.6%), *K. pneumoniae* (0.45%/0.42%/0.41%) and *Salmonella enterica* (0.02%/0.01%/0.02%).

### Dysbalanced SCFA-producing bacteria enable emerging of opportune pathogens

The significant changes (*p* ≤ 0.05; > threefold change) of the gut microbiota composition were identified on the family level of the bacterial taxonomy. The comparison of the individual time points showed a decrease in *Bifidobacteriaceae*, *Eubacteriaceae,* and *Erysipelotrichaceae* one week and one month after HSCT compared to samples before HSCT. Moreover, for samples taken one month after HSCT, depletion of *Lachnospiraceae*, *Oscillospiraceae*, *Coprococcaceae,* and *Clostridiaceae* was typical.

The comparison of individual time points revealed notable changes in the relative abundance of various bacterial families compared to healthy subjects. The decrease was observed in *Bacteroidaceae, Prevotellaceae, Oscillospiraceae, Bifidobacteriaceae, Eubacteriaceae, Odoribacteriaceae, Sutterellaceae,* and *Rikenellaceae* across all time points of sampling. Additionally, samples one week and one month after HSCT exhibited a depletion in *Erysipelotrichaceae* and *Lachnospiraceae*. *Akkermansiaceae* depletion was specifically observed before and one month after HSCT. Furthermore, the gut microbiota of patients one week after HSCT displayed a decrease in *Clostridiaceae*. On the other hand, there was a consistent increase in *Enterococcaceae* across all intervals. Notably, samples before HSCT also showed an increase in *Streptococcaceae*, while samples one week after HSCT exhibited an increase in *Staphylococcaceae* (Fig. 1).

Despite the significant decrease in certain bacterial families, the analysis identified bacterial representatives in individuals that potentially carry genes of antibiotic resistance, including *Akkermansiaceae* (*A. muciniphila*), *Lachnospiraceae* (*[Ruminococcus gnavus]*), *Clostridiaceae* (*C. difficile, Clostridium perfringens*). Furthermore, dominant bacterial families in the patient’s gut microbiome were also represented by resistant *Enterococcaceae* (*E. faecalis, E. faecium, E. hirae*), *Streptococcaceae* (*S. gallolyticus, S. infantis, S. agalactiae, S. suis*) and *Staphylococcaceae* (*S. aureus, S. epidermidis, S. hominis, S. haemolyticus*).

*Akkermansia muciniphila* was detected in all samples of healthy individuals (0.003%-7.3%) as well as in samples of patients except for one, one week after HSCT. Before HSCT no significant difference (*p* = 0.16) in relative abundance between patients with FN (0.0004%-0.02%) and neutropenia without fever (0.0003–12.3%) was detected, while one week after HSCT trend to higher relative abundance (*p* = 0.08) in the cohort of patients without FN (without FN 0.0003–0.06%; with FN 1.10^−12^− 0.007%) was identified. We have also investigated the change in relative abundance in each individual in the course of the therapy, but also no significant differences in the ratio before HSCT/one week after HSCT between the two cohorts of patients was found. Changes in the relative abundance of *Akkermansia* did not indicate the onset of fever during neutropenia in paediatric patients in conditioning regimen undergoing HSCT including in this study.

### Universal antimicrobial prophylaxis treatment spanning HSCT stumble upon gut microbiome resistome

Microorganisms carrying resistance genes can directly impact the efficacy of oncology treatments. They can activate prodrugs into their active forms, but there is also a risk of increased toxicity. Additionally, the gut microbiota can indirectly influence drug metabolism and toxicity by competing with bacterial-derived metabolites for xenobiotic metabolism pathways or modulating the host's metabolic systems^48^. The resistant bacteria could play a crucial role in these mechanisms through their possibility to survive antimicrobial prophylaxis.

Before HSCT, the resistome of the patients comprised a total of 57 different resistance genes (Supplementary Table 6). The most frequently found genes belonged to *aac(6')-Ii* detected in 8 patients, followed by *msr(C)* in seven patients, and *tet(W)* together with *sul2* in six patients. We have identified genes *aph(3″)-Ib, tet(O), erm(B), cfxA5, dfrG* in five patients, while *aac(6′)-aph(2″), ant(6)-Ia, aph(6)-Id, blaTEM-116* in 4 patients.

One week after HSCT, the prevalence of *aac(6′)-Ii* and *msr(C)* remained (eight patients), while *dfrG* and *blaTEM-116* emerged as the second most abundant ones (seven patients). The number of patients with *ant(6)-la* carried resistance increased by 50% (six patients), while *erm(T)*, *erm(B)* together with *aph(3')-III* were found in four patients. However, a significant decrease in *tet(W)* prevalence in the gut microbiome of patients was observed since this gene was detected exclusively in one gut microbiome sample one week and one month after HSCT.

Furthermore, *gene aac(6')-Ii* coding for chromosomal-encoded aminoglycoside acetyltransferase and aminoglycoside nucleotidyltransferase encoded by *ant(6)-la* gene were detected in seven samples, even one month after HSCT. We have identified gene *msr(C)* conferring resistance to macrolides in six patients, followed by dihydrofolate reductase encoded by the *dfrG gene* in five samples. Compared to the one week after HSCT, concerning *erm(B)* and *aph(3')-III* genes of resistance, we have identified no changes (four patients).

**Figure 4:**
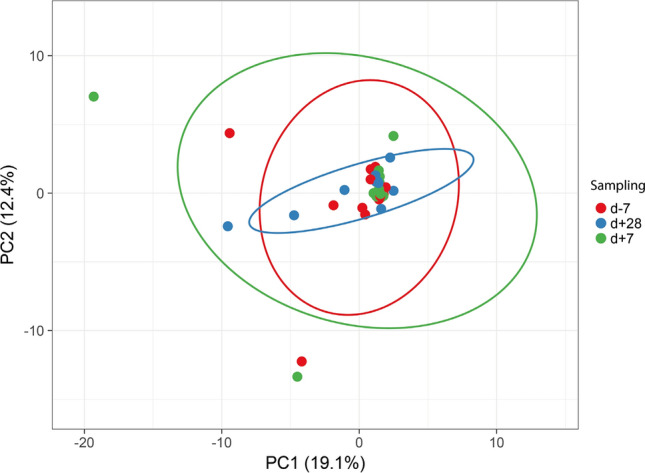
Beta diversity of analysed samples represented by the full resistome profile of gut microbiota of paediatric oncology patients before (d-7), one week after (d + 7) and four weeks after (d + 28) HSCT. Resistome was represented by a set of antimicrobial resistance genes identified within the analysed sample. SVD with imputation was used to calculate the principal components. The X and Y axis show principal component 1 and principal component 2 that explain 19.1% and 12.4% of the total variance. Prediction ellipses represent 0.95% probability for new observation to fit the group (n = 47).

Based on the additional statistical tests, we have found *cfxA5* and *tet(W)* more often in the gut microbiota of patients before HSCT. At the same time, *mef(A)* was identified only one week after HSCT in three of 16 patients (*p* < 0.05). Furthermore, *tet(W)* significantly differed between samples, detected more often before HSCT (38% of patients before HSCT, 6% one week after HSCT). We have not detected any significant changes in gut resistome one week and one month after HSCT. These results indicate a higher abundance of specific antimicrobial resistance genes before HSCT, suggesting a potential association of the conditioning regimen with the occurrence of resistant bacteria (Fig. 4).

The frequency of resistance genes in the identified resistant bacteria varied across different bacterial species. *K. pneumoniae* exhibited the highest number of resistance genes with a total of 23 genes, including *blaTEM-1C, blaTEM-1B, blaTEM-181, aph(3")-Ib, sul2, aac(3)-IIa, aph(6)-Id, aac(6')-Ib-cr, aadA16, tet(D), blaSHV-1, blaOXA-1, blaCTX-M-15, OqxA, qnrB1, dfrA14, qnrB6, dfrA27, ARR-3, OqxB, fosA, aph(3')-Ia, catA1*). 16 resistance genes have been observed in *E.coli* (*blaTEM-33, blaTEM-169, aac(3)-IV, aph(4)-Ia, tet(A), blaTEM-1C, sul1, blaTEM-116, aph(3'')-Ib, aph(6)-Id, aadA1, tet(B), blaCTX-M-1, blaTEM-1B, dfrA1, sul2*) and 13 genes in *E. faecium* (*aac(6')-Ii, erm(T), msr(C), VanHAX, cat, dfrG, ClpL, tet(X), aph(3')-III, aac(6')-aph(2''), ant(6)-Ia, erm(B), tet(S)*). *S. haemolyticus* carried 11 resistance genes (*aac(6')-aph(2"), aph(3')-III, tet(K), blaZ, mecA, msr(D), msr(A), mph(C), mef(A), qacA, dfrG*) and ten various genes have been carried by *B. fragilis* (*sul2, tet(X), tet(Q), erm(F), cfxA3, cfxA, cfxA5, cfxA4, cepA, cepA-29*). Resistance to β-lactams, aminoglycosides, and streptogramin b was the most frequently observed in these bacteria.

Resistance to tetracyclines, β-lactams, unknown β-lactams, and a folate pathway antagonist in controls was encoded by ten identified resistance genes. All eight controls, with identified resistance genes, exhibited the presence of tetracycline resistance genes. The specific resistance genes were *tet(Q)* in six out of eight controls, *tet(O)* (1/8), *tet(W)* (5/8) encoding ribosomal protection proteins, and *tet(X)* (1/8) encoding direct enzymatic inactivation of the drug were present in all eight controls^49^. Tetracycline resistance spreads rapidly among bacteria because of the localization of tet genes on plasmids, transposons, and integrons^50^. In two controls, the *tet* genes were located on transmissible elements, *tet(Q)* was identified in *B. fragilis* conjugative transposon CTn341 and *tet(W)* in *Cutibacterium acnes* TP-CU389 plasmid pTZC1. Three healthy individuals had also detected resistance genes of β-lactams and unknown β-lactams (*cfxA* (2/8), *cfxA3* (2/8), *cfxA4* (2/8), *cfxA5* (2/8), *cfxA6* (1/8)). Folate pathway antagonist was detected only in one control (*sul2* gene).

### Antimicrobial genes in genomes of opportune pathogens and commensal bacteria

The contigs containing identified resistance genes were analysed using the BLAST tool to assign taxonomic classification and determine their origin within bacterial strains.

Our study identified fifty-two bacterial genera and five plasmid vectors carrying antibiotic-resistance genes. In healthy children, mostly commensal bacteria carried antimicrobial resistance. These bacteria involved members of phylum *Bacteroidota* (*Parabacteroides* spp., *Bacteroides* spp., *Phocaeicola* spp., *Chryseobacterium* spp.) but also *Bacillota* (*Clostridium* spp., *Dysosmobacter* spp.) or *Actinomycetota,* represented by *C. acnes* as the only identified species. The gut microbiota of the patients was rich in resistance genes observed mainly in opportunistic pathogens such as *E. faecium, E. faecalis, E. coli, K. pneumoniae, E. hormaechei, S. enterica, S. aureus, S. haemolyticus, C. difficile* or *Campylobacter coli*.

The comparison of the individual collections showed that resistant *E. faecium* was present in patients consistently throughout the observation period (8/8/7), whereas *E. faecalis* was detected in one more patient after HSCT (1/2/2). *E. coli* was least abundant before HSCT, increasing with time since HSCT (2/3/5). More patients carried resistance genes in *K. pneumoniae* (5/2/2), *B. fragilis* (4/0/1), *P. distasonis* (3/0/0), *S. enterica* (2/1/0), or *S. suis* (2/0/1) before HSCT compared to the post-HSCT period. Post-transplant patients had an increased abundance of resistant bacteria represented by *E. hormaechei* (0/0/2), *S. pneumoniae* (0/1/1), *S. gallolyticus* (1/2/1), or *S. haemolyticus* (0/1/2).

**Figure 5:**
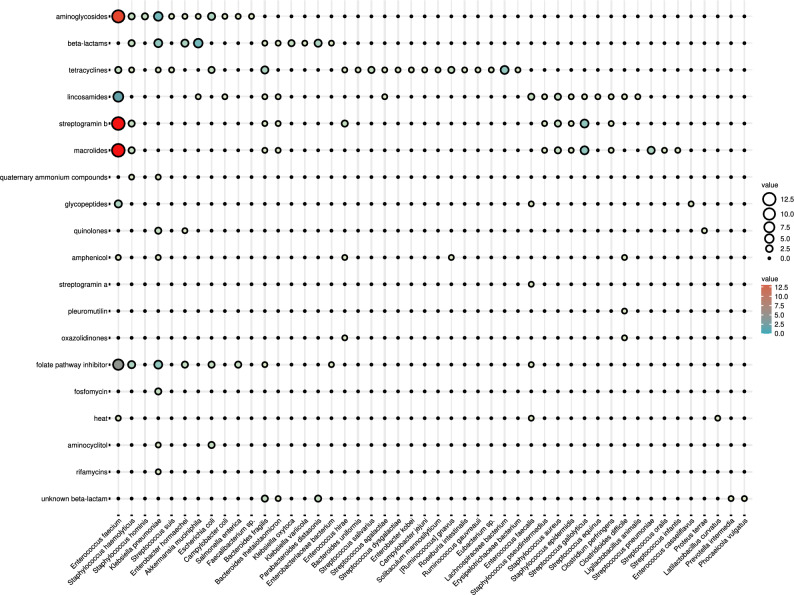
Graphical visualization of the absolute number of antimicrobial resistance genes clustered according to their predicted phenotype carried by bacterial genera detected in gut microbiome of healthy individuals and patients.

A closer analysis of patients with FN showed that in the period before HSCT, these patients had resistance genes more frequently carried by *Enterococcus* (*E. faecium, E. faecalis, E. hirae*), *Staphylococcus* (*S. aureus, Staphylococcus pseudintermedius*), *Bacteroides* (*B. fragilis, Bacteroides thetaiotaomicron*) *K. pneumoniae*, *Streptococcus* (*S. gallolyticus, S. suis*) *S. enterica*, and *C. difficile* compared to patients without FN Patients without FN had resistance genes carried by *S. agalactiae, P. distasonis, Enterobacteriaceae* bacterium, *A. muciniphila, C. coli*, and *C. perfringens*. One week after HSCT, *E. faecium* and *E. faecalis* were identified with higher prevalence in patients compared to samples before HSCT, which suggested an increase in the abundance of these species following HSCT. Additionally, during the same time, these species were more prevalent in patients with FN In patients with FN, resistance genes were observed in *Streptococci* species (*S. gallolyticus, S. pneumoniae, S. agalactiae*), *E. coli*, and *S. enterica*. In contrast, patients without FN had genes carried by *S. aureus.* After one month, bacteria of the genera *Enterococcus*, and *Bacteroides*, together with species *K. pneumoniae*, *S. gallolyticus*, and *E. bacterium*, carried resistance genes in patients with FN *S. haemolyticus, S. epidermidis, S. pneumoniae, S. suis, S. infantis, A. muciniphila, E. coli,* and *C. coli* carried resistance genes one month after HSCT in the group of patients without FN (Fig. 5).

### Genotypic prediction of phenotypic resistance to antimicrobials of gut bacteria

A total of 84 genes were identified within 20 detected groups of antimicrobial resistances. From 14 healthy controls, only eight individuals carried genes for antimicrobial resistance. 8/14 (57%) of all healthy individuals were resistant to tetracyclines; in 3/14 (21%), resistance to β-lactams was recorded, and in 1/14 (7%), resistance to unknown β-lactams and folate pathway antagonists were detected.

Eighty-three of these genes were identified in the group of patients. Each patient showed a different number and combination of identified resistance genes at every time point of sample collection. Genes providing aminoglycoside resistance were identified in each patient (100%), followed by lincosamides (89%), streptogramin b (89%), macrolides (89%), folate pathway antagonist (89%), β-lactams (83%) and tetracyclines (78%). The resistome of patients also included genes for unknown β-lactams, glycopeptides, amphenicol, oxazolidinone, heat resistance, quinolones, quaternary ammonium compounds, antibacterial steroids, streptogramin a, pleuromutilin, fosfomycin, aminocyclitol, and rifamycin; less than 50% of patients carried the genes mentioned above.

The predicted phenotype of the resistance genes (Supplementary Table 7) involved 18 antimicrobial groups detected in the samples before HSCT, 17 one week, and 15 one month after HSCT (16 samples before HSCT/16 samples one week after HSCT/14 samples one month after HSCT). Resistance to aminoglycosides prevailed the most before and after HSCT (12/11/10), followed by tetracyclines (11/7/4), which exhibited a decreasing trend. β-lactam (8/8/8) and macrolide resistance (9/9/9) were consistently present in gut microbiota, together with lincosamides (10/10/9), streptogramin b (9/9/10), and folate pathway antagonist (11/8/8). Unknown β-lactams were mainly detected before HSCT (6/1/1). The less often was resistance to glycopeptides (2/1/3), amphenicol (4/2/1), quinolones (3/2/2), quaternary ammonium compounds (1/3/1), and aminocyclitol (1/2/1) varying and fluctuating between patients. The gut microbiota of one patient carried resistance to heat and fosfomycin at all time points (1/1/1). Finally, predicted resistance to oxazolidine and pleuromutilin was detected only before HSCT, while rifamycin and antibacterial steroids were identified only one week after HSCT. Of all predicted antimicrobial groups, resistance to tetracycline and unknown β-lactams encoded by gut microbiota was significantly higher before HSCT compared to the period after HSCT (*p* ≤ 0,05) (Fig. 5.)

**Figure 6:**
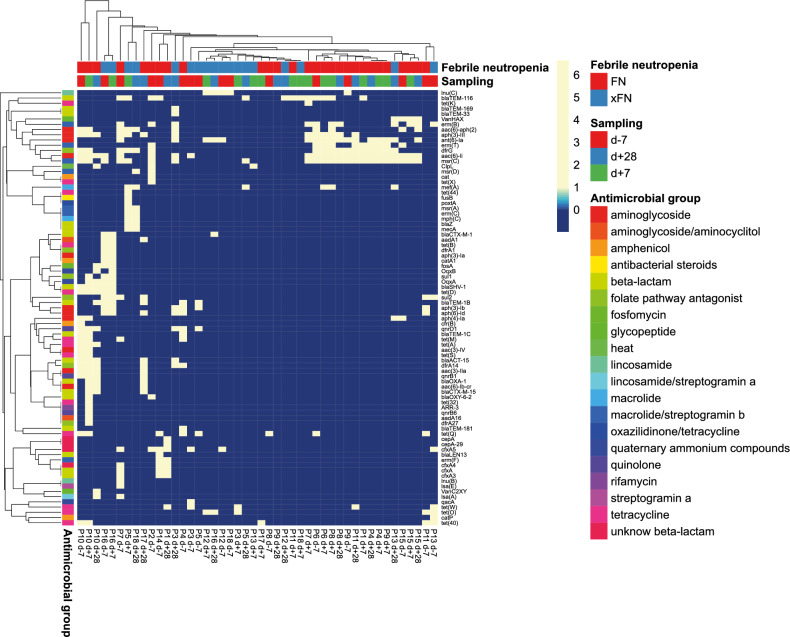
Graphical visualization of the spread of antimicrobial resistance genes annotated by the predicted antimicrobial group phenotype in paediatric oncology patients before (d-7), one week after (d + 7) and four weeks after (d + 28) HSCT with focus on febrile neutropenia outcome after HSCT (FN – patients with febrile neutropenia, xFN – patients without febrile neutropenia). Rows are centred; unit variance scaling was applied to rows. Both rows and columns were clustered using correlation distance and Ward linkage.

Although there was no overall typical resistome profile of the gut microbiota before HSCT and after HSCT, a set of antimicrobial resistance genes associated with FN has been identified. The resistome before HSCT included a distinct set of genes (*msrC, VanHAX, erm(B), aac(6)-aph(2), aph(3)-III, ant(6)-Ia, erm(T), dfrG, aac(6)-Ii*), which were found to discriminate the group of patients with FN (Fig. 6).

### Gut bacteria associated with febrile neutropenia through antimicrobial resistance genes

The patients who developed FN (Supplementary Table 8) exhibited a distinct pattern of resistance genes (Fig. 6), specifically genes *VanHAX*, *erm(B)*, *aac(6)-aph(2)*, *aph(3)-III*, *ant(6)-Ia*, *erm(T)*, *dfrG*, *aac(6)-Ii*, *msr(C)* along with the presence of *blaTEM-116* and *mef(A)*. Within the group of patients developing FN, the gut resistome before HSCT possessed common features (genes of antibiotic resistance) in 60% of patients, while after HSCT the number of patients with similar resistome increased to 80% based on Pearson correlation (Fig. 6). Using.

**Figure 7:**
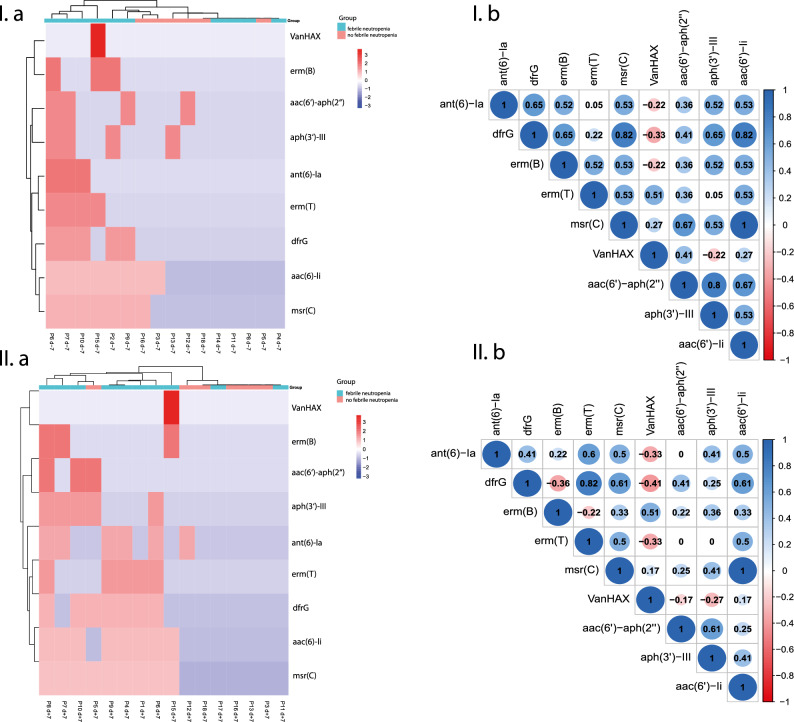
Subset of biomarkers—antimicrobial resistance genes detected in gut microbiome of patients who developed febrile neutropenia, before HSCT (I.a) and after HSCT (I.b) and spearman rank correlation analysis of selected genes (I.b – before HSCT; II.b – one week after HSCT) graphically visualized by a correlation matrix. Correlations ≥ 0.6 (p ≤ 0.05) were considered for further evaluation. (I.b; d-7) who developed febrile neutropenia after HSCT.

Spearman rank correlation analysis we have identified a subset of genes significantly (*p* ≤ 0.05) correlating including *aac(6')-Ii / drfG / msr(C)* (*p* = 1.10^–11^− 0.004), *drfG* with *ant(6)-Ia*, *erm(B)*, *aph(3')-III* (*p* = 0.04) and *aac(6')-aph(2'')* with *msr(C)*/*aac(6')-Ii* (*p* = 0.04) in gut resistome before HSCT. Despite higher number of patients with similar resistome, one week after HSCT, significant correlation has been detected only between *erm(T)*/*drfG* (*p* = 0.004) and *msr(C)*/*aac(6')-Ii* (*p* = 1.10^–12^) (Fig. 7).

Before HSCT, the triple combination of *aac(6')-Ii/drfG/msr(C)* genes was chromosomally localized in different strains of *E. faecium* including *E. faecium* ME3, *E. faecium* E6043, *E. faecium* KUHS13, *E. faecium* E8377, *E. faecium* ICU-5-1, *E. faecium* WE0851, *E. faecium* 4928STDY7071735. *dfrG* together with *ant(6)-la* were detected on the chromosome and on plasmid of different *E. faecium* strains (P6; *E. faecium* ME3 and *E. faecium* ICU-1-2 plasmid pZA1-2_03), on chromosome of different bacterial genera (P7; *E. faecium* WE0851, *Streptococcus suis* SC317), and colocalized in one genome of *E. faecium* (P9; *E. faecium* KUHS13). Cooccurrence of genes *dfrG* with *erm(B)* was detected in 4 patients also mainly in the on the chromosome of *E. faecium* and *S. pseudintermedius* (P2; *dfrG*- *E. faecium* ME3; *erm(B)*- *Staphylococcus pseudintermedius* MAD627); but also on different strains of *E. faecium* plasmid (P6; *dfrG*- *E. faecium* ME3, *erm(B)*- *E. faecium* ICU-1-2 plasmid pZA1-2_03); (P7; *dfrG*- *E*. *faecium* WE0851, *erm(B)*- *E. faecium* CVM N60443F plasmid pN60443F-1) and P10 (*dfrG*- *E*. *faecium* KUHS13, *erm(B)*- *E. faecium* AALTL plasmid pEFA-790c). The combination of *dfrG* with *aph(3’)-III* was observed in P6, where *aph(3’)-III* was identified on *E. faecium* ICU-1-2 plasmid pZA1-2_03 and P7 with the *aph(3’)-III* in *E. faecium* E8440.

The *erm(T)* / *drfG* combination one week after HSCT was detected in two different bacterial genera *Enterococcus* and *Staphylococcus* (P1; *erm(T)—E. faecium* ME3, *dfrG*—*Staphylococcus haemolyticus* SH9361), or *Enterococcus* and *Streptococcus* (P8/P9; *erm(T)*—*Streptococcus gallolyticus* FDAARGOS_666, *dfrG*—*E. faecium* ME3 (P8) and *E. faecium* KUHS13 (P9)), two strains of *E.faecium* (P4; *E. faecium* ME3/*E. faecium* KUHS13) and within the same genome of *E. faecium* ME3 (P6). However, cooccurrence of genes *msr(C)*/*aac(6')-Ii* was associated exclusively with *E.faecium* strains: P1 (*aac(6′)-li*—*E. faecium* K-189-3; *msr(C)*—*E. faecium* ME3), P4 (*aac(6′)-li*—*E. faecium* ME3; *msr(C)*—*E. faecium* KUHS13), P7 (*aac(6′)-li*—*E. faecium* isolate E8377; *msr(C)*—*E. faecium* ICU-5-1), P9 (*aac(6′)-li*—*E. faecium* ME3; *msr(C)*—*E. faecium* isolate E6043) and P15 (*aac(6′)-li*—*E. faecium* ICU-1-2; *msr(C)*—*E. faecium* VVEswe-R); (P6/P8; both genes in *E. faecium* ME3), (P10; both genes in *E. faecium* E6043).

Detected genes were mainly present in the *E. faecium* strains. Moreover, *erm(B)* was also detected in *Staphylococcus pseudintermedius* and *E. faecalis* strain CVM N60443F, while *aac(6)-aph(2)* in *S. hominis* strain FDAARGOS_661 and *erm(T)* in *S. gallolyticus* strain FDAARGOS_666. Correlation analysis revealed that the resistome of most patients with FN clustered together, suggesting a potential association between the gut microbiome resistome and FN as one of the HSCT outcomes (Fig. 7). Our finding implies that these genes may serve as markers indicating the onset of FN.

For the rest of patients with FN, another distinct cluster of gut microbiota resistome characterized by a more diverse profile of resistance genes including *blaCTX-M-1*, *aadA1*, *tet(B)*, *dfrA1*, *aph(3)-Ia*, *catA1*, *fosA*, *OqxB*, *sul1*, *OqxA*, *blaSHV-1*, *tet(D)*, *sul2*, *blaTEM-1B*, *aph(3)-Ib*, *aph(6)-Id*, *tet(M)*, *tet(A)*, *aac(3)-IV*, *tet(S)*, *blaACT-15*, *dfrA14*, *aac(3)-IIa*, *qnrB1*, *blaOXA-1*, *aac(6)-Ib-cr*, *blaCTX-M-15* and *blaOXY-6-2* was identified.

On the other hand, it was evident that the gut microbiota resistomes of patients who did not develop FN also clustered and exhibited significantly lower diversity or absence of resistance genes than those with FN. This observation suggests that patients with FN may possess a broader range of resistance genes, potentially correlating with the development of FN. Different clusters of the resistance genes may serve as biomarkers with potential in personalized approaches.

The more detailed analysis made identifying multidrug-resistant bacteria in the individual patient's sample possible. The patients who developed FN carried more resistance genes associated with the opportunistic pathogens *Enterococcus* spp., *Klebsiella* spp., and *Staphylococcus* spp. in their gut microbiome before HSCT. The prevailing *E. faecium* strains (*E. faecium* ME3, *E. faecium* KUHS13, *E. faecium* ICU 1-2, *E. faecium* VVEswe-S) carried variable combinations of resistance genes *msr(C)*, *erm(T)*, *dfrG, erm(B)*, *aac(6)-li*, *ant(6)-la*, *aph(3)-III* and *VanHAX*, located both on the chromosome and on the plasmids. We noticed *K. pneumoniae* strains (*K. pneumoniae* B16KP0141, *K. pneumoniae* QD23, *K. pneumoniae* 21) being a significant source of resistance genes carrying *blaTEM1C* / *blaTEM1B* / *blaTEM-181* genes, *OqxA*/*OqxB* genes or *sul2*/*sul1* genes. However, individual evaluation of gut microbiome revealed also *K. pneumoniae* C16KPO160 strain carrying *tet(A)*, *aadA16*, *ARR-3*, *dfrA27*, and *aac(6')-Ib-cr* one week after HSCT.

The patients without FN had more genes encoding resistance to antibiotics carried by multidrug-resistant opportunistic pathogens, mainly after HSCT (one week or one month after HSCT). In comparison, before HSCT and seldom also one week after HSCT, the multidrug-resistant bacteria belonged to commensals (*B. fragilis*, *P. distasonis*, [*Ruminococcus*] *gnavus*).

Before HSCT, earlier occurrence of resistance genes suggests a link between the earlier invasion of potentially pathogenic resistant bacteria and the FN outcome. Furthermore, the gut microbiota of patients without post-transplant complications encompassed multidrug-resistant bacteria mainly after HSCT, indicating an association between the time of the emergence of multidrug-resistant gut bacteria and FN. These findings highlight the importance of understanding the role of a subset of resistance genes, or the whole resistome, in the context of a patient's complication development and could provide insight for a more personalized approach to patient treatment.

### Common inflammation parameters and gut microbiome

Specific bacterial families, including *Enterococcaceae*, *Streptococcaceae*, and *Staphylococcaceae*, are commonly found in human gut. However, they can transform into pathobionts capable of translocating across the gut barrier into the blood resulting in bloodstream infections. This transition from commensals to opportunistic pathogens is a significant concern, particularly for immunocompromised individuals, who are at higher risk of infection. Moreover, these bacterial families have been identified as reservoirs of antibiotic resistance genes, further complicating their clinical outcome. The members of these bacterial families were often associated with higher mortality after HSCT and FN as well as GvHD development^51–53^.

**Figure 8:**
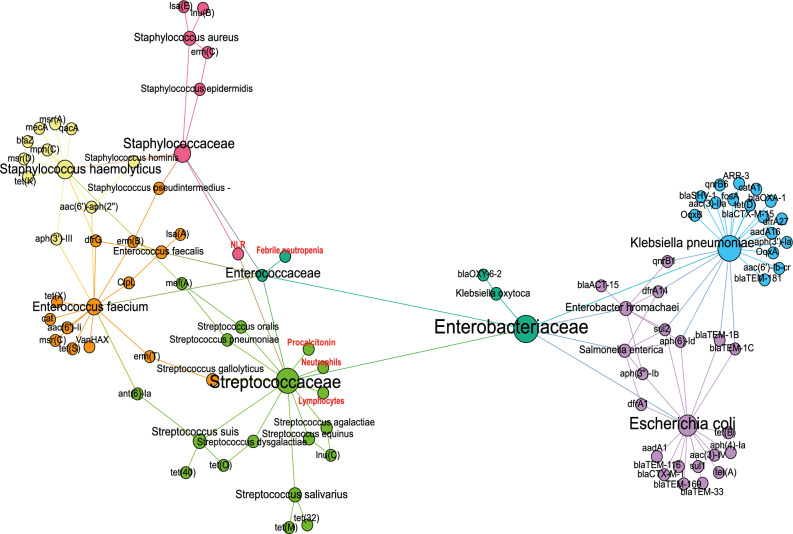
The network analysis visualizing the association between opportunistic pathogens *Enterococcaceae*, *Streptococcaceae, Staphylococcaceae,* antimicrobial genes of resistance, inflammation biomarkers CRP and procalcitonin and febrile neutropenia.

We aimed to investigate the relationship between available inflammation parameters (Supplementary Table 9) included in routine analysis and resistant bacterial taxa before and after HSCT. We looked for potential links between gut bacteria and fever as a stimulus, but our analysis did not reveal any differences in CRP levels between patients with or without FN. Moreover, CRP did not show any correlation to any bacteria. However, we did find a positive correlation between *Streptococcaceae* and procalcitonin (PCT) levels before HSCT, suggesting a potential link between these bacteria and systemic inflammation. Interestingly, one week after HSCT, a distinct pattern emerged where *Streptococcaceae* were negatively correlated with lymphocyte and neutrophil counts. These findings shed light on the complex interplay between gut bacteria and inflammation and could help to stimulate future research on treatment and prevention of FN. It appears that there is a potential relationship between the loss of lymphocytes and increased abundance of *Streptococcus*^54^, which could be a predictor of infection development. In addition, there is evidence that *Enterococcaceae* may be involved in the development of FN during the post-transplant period. Based on Spearman rank correlation, one month after HSCT, there was a notable link between *Streptococcaceae* and *Staphylococcaceae* and the neutrophils to lymphocytes ratio (NLR), suggesting their potential role in driving an imbalanced immune response. These findings highlight the complex interactions between specific gut microbiota members and the host, particularly in immunocompromised patients (Fig. 8). However, other bacterial families that carry resistance genes were not significantly correlated with clinical parameters.

### Personal gut resistome of patients can be associated with cancer treatment outcome

Patients undergoing cancer treatment that included antibiotics before and after HSCT exhibited resistance to specific antibiotic drugs. Biseptol (trimethoprim / sulfamethoxazole), Amikacin, and Tazocin (piperacillin / tazobactam) dominated among the antibiotics prescribed before HSCT, while after HSCT, Amikacin, Tazocin, and Targocid (teicoplanin) mainly were prescribed. Of all patients treated with antibiotics (18) mentioned earlier, resistant bacteria were found in the gut microbiome of 13 of them. More importantly, most of them, ten patients, developed FN after HSCT. Closer investigation of the gut microbiome of treated patients revealed resistance to Biseptol (trimethoprim/sulfamethoxazole; eight out of 10 patients treated; 80%) and Tazocin (Piperacillin/Tazobactam; 4 out of 7 patients; 57%) before HSCT, while resistance to Amikacin (Amikacin; three out of five patients; 60%) and Tazocin (four out of nine patients; 44%) after HSCT. Even though each patient sustained a different portfolio of bacteria in the gut microbiome, each carrying different resistance genes, the resistome of gut bacteria represented the standard feature. Bacteria-carrying genes for resistance to trimethoprim/ sulfamethoxazole provided by *drfG*, *drfA1*, *dfrA14*, and *sul2*, were identified exclusively before HSCT. The *dfrG* gene carried by *Enterococcus* spp. was detected in the gut microbiome of two patients, the *dfrA1* gene, carried by *S. enterica*, in one individual, and *S*. *enterica* together with *K. pneumoniae* encoded resistance to trimethoprim / sulfamethoxazole by *dfrA14* gene in two individuals. Both microbes, identified in the gut microbiome of four other patients, also possessed *sul2*-encoded resistance. *B. fragilis* was the only commensal bacteria predicted to provide resistance to trimethoprim/sulfamethoxazole in one patient.

The genes *aac(6')-aph(2"), aph(3')-III* carried in the genomes of *E. faecium* and *S. hominis*, and *aac(6')-Ib-cr* in *K. pneumoniae* encoded resistance to Amikacin and *VanHAX* gene in *E. faecalis* could contribute to Teicoplanin resistance. Mainly in the genome of *E. coli*, but also *Klebsiella* spp., we have identified seven *bla* genes encoding resistance to piperacillin/tazobactam (Table 1). Among them, *blaTEM-116*, carried by unidentified bacteria and *E. coli*, prevailed (6 patients).

**Table 1:** Identified resistance to antibiotics used by patients before and one week after HSCT. Resistance genes carried by bacteria to listed antibiotics used by patients.

Antibiotics	Gene	Microorganism
D-7		
Trimethoprim/sulfamethoxazole	*dfrG*	*Enterococcus faecium*
		*Enterococcus faecalis*
	*dfrA14*	*Salmonella enterica*
		*Klebsiella pneumoniae*
	*sul2*	*Salmonella enterica*
		*Klebsiella pneumoniae*
		*Bacteroides fragilis*
	*dfrA1*	*Salmonella enterica*
Amikacin	*aac(6′)-aph(2″)*	*Enterococcus faecium*
Piperacilline/tazobactam	*blaTEM-116*	unknown
	*blaOXY-6-2*	*Klebsiella oxytoca*
	*blaTEM-1C*	*Escherichia coli*
	*blaSHV-1*	*Klebsiella pneumoniae*
	*blaLEN13*	*Klebsiella variicola*
D + 7		
Amikacin	*aph(3')-III*	*Enterococcus faecium*
	*aac(6′)-aph(2″)*	*Staphylococcus hominis*
		*Enterococcus faecium*
	*aac(6')-Ib-cr*	*Klebsiella pneumoniae*
Teicoplanin	*VanHAX*	*Enterococcus faecalis*
Piperacilline/tazobactam	*blaTEM-116*	unknown
		*Escherichia coli*
	*blaSHV-1*	*Klebsiella pneumoniae*
	*blaTEM-1B*	*Escherichia coli*
	*blaCTX-M-1*	*Escherichia coli*

Potential antibiotic resistance was encoded by six bacterial genera—*Enterococcus*, *Staphylococcus*, *Klebsiella*, *Escherichia*, *Salmonella*, and *Bacteroides*. Most of the resistance genes were carried by *K. pneumoniae* and *E. coli* (Table 1).

It has been found that certain bacterial species can have an impact on cancer therapy. The Pharmacomicrobiomics and Microbiota-Active Substance Interaction Database (MASI) were searched to identify bacterial species carrying antimicrobial resistance genes. Within the gut microbiome of all patients, only resistant bacteria *B. fragilis* and *R. intestinalis* were found to have a positive interaction with the anticancer drug methotrexate (MTX), decreasing its toxicity. *Prevotella*, on the other hand, was associated with increased drug activity.

It is interesting to notice, that only three of the thirteen patients who received MTX showed the presence of these particular bacteria in their gut microbiome, while two of them developed FN: one patient with two strains of *B. fragilis* (*B. fragilis* DCMOUH0018B, *B. fragilis* S14) and one patient with *B. fragilis* FDAARGOS_763. Conversely, the in the gut microbiome of the third patient who did not exhibit FN *B. fragilis* BFG-79 and *B. fragilis* DCMOUH0017B, as well as *R. intestinalis* L1-82 were identified. Although we did not identify any pattern, these preliminary results show importance of considering also commensal bacteria carrying genes of antimicrobial resistance as important players also in drug metabolism. However, their potential needs to be further elucidated.

## Discussion

The process of HSCT profoundly impacts the microbial composition and diversity in the gut. The gut microbiome of paediatric oncology patients changes due to the conditioning regimen, that can include chemotherapy or total body irradiation. Usually, the presence of commensal bacteria prevents colonization of the gut by pathogens. However, the use of antibiotics can alter not only the gut microbiota leading to an imbalance known as dysbiosis but also causes changes in the gastrointestinal barrier^55^. It has been observed that a decrease in gut microbial alpha-diversity is linked to higher mortality rates in patients undergoing allo-HSCT^56,57^ or in correlation to GvHD associated mortality^58^. The investigation by Masetti et al. (2022) found a consistent decline in diversity among patients with extended fever duration during the post-HSCT timeframe^17^. Similarly, Rattanathammethee et al. (2020) also noted a significant decrease in alpha-diversity during the fever neutropenic phase^59^. These findings indicate that altered gut microbial diversity may have potential implications on patient outcomes.

Standard treatment protocols containing broad-spectrum antibiotics lead to an increase in the number of resistant bacteria^60^. Moreover, the patients are more susceptible to fungal or bacterial infections, including *Klebsiella* spp., *Enterococcus* spp., *Staphylococcus* spp., and *Enterobacter* spp. as the infectious agent^61^. The presence of opportunistic pathogen bacteria is a matter of significant concern. This study identified bacteria in a considerable proportion of the gut of HSCT patients, highlighting the ones with genomes enriched in resistance genes with a possible impact on the therapy. The emergence of resistant strains could be attributed to several factors, but one of the most likely culprits is prolonged antimicrobial therapy. This is often part of the prophylaxis for patients undergoing HSCT and the suppression of the immune system. *E. faecium* was consistently the most common bacterial species found in the gut microbiome of patients, regardless of the time intervals studied. Based on the available data, it appears that the increased abundance of the genus *Enterococcus* is a significant factor in the gut microbiota of patients undergoing HSCT^62–65^. Enterococci belong to opportunistic pathogens of the human gut microbiota and are known for their multi-resistance, which can pose a significant problem in healthcare. It is important to note that in patients undergoing HSCT, *Enterococcus* has been found to have a positive correlation with both mortality and the development of GvHD. The study of Smith et al. (2022) highlighted *Enterococcus* as an indicator of clinical outcomes^66^. *E. faecium* was not only the most prevalent bacteria in the cohort of patients but was identified as the primary carrier of resistance genes in the majority of patients; however, it was not observed in only three patients.

The depletion in beneficial SCFA-producing bacteria, such as *Bacteroidaceae*, *Oscillospiraceae*, or *Eubacteriaceae*, was detected through all the samples. It has been found that a reduction in SCFA in patients can have a significant impact on their health. This can lead to changes in glucose homeostasis, gut integriy, and immunomodulation^67^, which in turn increases the risk of bloodstream infection^68^. It is important to note that SCFAs have been extensively researched regarding their implications for GvHD, where they have been shown to protect against the development of chronic GvHD. Conversely, the depletion of anti-inflammatory members of the *Clostridia* class has been associated with an increased risk of developing these complications^69^. Interestingly, *Akkermansiaceae* were depleted in the gut microbiota of patients before and one month after HSCT. On the contrary, one week after HSCT, the patient's gut microbiota showed a higher relative abundance of *Akkermansiaceae* compared to healthy subjects. The most known member, *A. muciniphila*, has been shown to play a crucial role in maintaining the integrity of the gut barrier^47^ while also breaking down mucin^70^. *A. muciniphila* has been found to be more prevalent in patients with inflammatory diseases like inflammatory bowel diseases^71^, which could indicate an increased effort to repair the intestinal barrier. Furthermore, *Akkermansia* expansion in the gut of patients with acute leukaemia predicted a higher risk for neutropenic fever, probably through the regulation of microbiota-host metabolic interaction by modulating the mucosal interface^72^. The patients were often found with an injured gut barrier that can lead to bacteria translocation and system infection, which can ultimately result in sepsis. Monitoring the microbial composition and resistance occurrence in HSCT patients is crucial due to their suppressed immune system, which can result in neutropenia and a significant reduction in the number of neutrophils responsible for fighting bacterial infections. Even with broad-spectrum antibiotics, resistant microorganisms can still grow, as evidenced by our results and other studies. Opportunistic pathogens like *E. faecium* have been confirmed to persist even after antibiotic prophylaxis, leading to their dominance and, in some cases, monodominance. It is interesting to note that in our study, in the gut microbiota of patients undergoing HSCT, both with and without FN, *E. faecium* dominated the gut bacterial resistome.

As expected, the patient's resistance profile differed from that of healthy individuals carried by commensal bacteria. In the healthy subjects, the *tetQ* gene conferred the highest prevalence of resistance to tetracyclines, followed by resistance to unknown β-lactams, β-lactams, and sulfamethoxazole.

We have discovered the significant role, particularly of the *Bacteroides* strains. These strains possess a remarkable resistance to β-lactams and tetracyclines, thanks to the crucial involvement of genes such as *cfxA*, *cfiA*, *cepA*, and *tetQ*^73^. Bacteria can acquire resistance genes through mutations that spread vertically during cell division or through the horizontal gene transfer process, where resistance genes are carried on mobile genetic elements. These horizontally acquired genes can further spread vertically, contributing to the spread of resistance genes in microbial ecosystems like the gut microbiota^74^. It is worth noting that up to 80% of *Bacteroides* spp. isolates contain a CTnDOT conjugative transposon that carries the *tetQ* gene and *ermF*, a macrolide-resistance gene^75^.

Additionally, we have discovered different clustering patterns of resistomes of patients with FN prevalently carried by *E. faecium*, mainly resistant to aminoglycosides. It is interesting to note that Enterococci have an intrinsic low resistance to aminoglycosides because of their relative impermeability to aminoglycoside antibiotics that are associated with species-specific chromosomal *aac(6′)-Ii* gene encoding a 6'-N-aminoglycoside acetyltransferase^76^. ESKAPE is a group of pathogens responsible for most hospital infections. These pathogens, including *E. faecium*, *S. aureus*, *K. pneumoniae*, *Acinetobacter baumannii*, *Pseudomonas aeruginosa*, and *Enterobacter*, are becoming increasingly resistant to antibiotics, such as aminoglycosides^77^. Among the gram-negative bacteria, *aac(6′)-Ib* is one of the most common^78–80^. Because of its localization on transposable elements, plasmids, or phages, based on the ability of these genetic elements to spread, theoretically, it can be acquired by any gram-negative bacteria. Our results show that gram-negative *Enterobacteriaceae*, specifically *K. pneumoniae* and *E. coli*, were prevalent resistant bacteria within the patient group and carried genes encoding resistance primarily to β-lactams and aminoglycosides. These bacterial species are frequently isolated from patients with FN^76,81^. However, high-level resistance to aminoglycosides mediated by aminoglycoside-modifying enzymes has been increasing in recent years. These enzymes are encoded by several aminoglycoside resistance genes, including *aac(6′)-aph(2′′), aph(3′)-III, ant(6′)-Ia,* and *aph(2′′)-Ib*^82^. Historically, *aac(6′)-aph(2′′)* was thought to be restricted only to gram-positive enterococci and staphylococci; nowadays, it was detected in *Campylobacter*, *Streptococcus*, *Clostridium perfringens*, or *Lactococcus garvieae*. Together with *aph(3′)-III* commonly found in *S. aureus* and *E. faecalis*, *ant(6′)-Ia* detected either on plasmid or chromosome *of S. aureus, S. epidermidis*, *E. faecium* and *E. faecalis*, as well as *Streptococcus mitis, S. suis*., and *aph(2′′)-Ib* of *E. faecium* and *E*. *coli* they encode all three types of aminoglycoside modifying enzymes^83^. Specifically, in the gut microbiome of patients undergoing HSCT, we have identified *aac(6')-aph(2'')* in the genome of *E. faecium* encoding bifunctional aminoglycoside-inactivating enzymes that possess activity from both enzyme components, thereby providing resistance to the combination of antibiotics; it may include acetylation, phosphorylation or nucleotidylation activity. In other *E. faecium*, *aph(3')-III* encoding O-phosphotransferase responsible for resistance to kanamycin and neomycin was identified. Within the *Enterobacteriaceae* family, a specific type of *aac(6')-Ib, aac(6')-Ib-cr* variant can induce double resistance to aminoglycosides and fluoroquinolones. Except for *E. coli*^84^ and *Enterobacter*^85^, also in blood isolates, *K. pneumoniae* has been identified as a carrier of this type of resistance^86^. These findings correlate with our results, including an increased abundance of *Enterococcus* spp. that has been observed in the gut microbiome of patients post-HSCT and suggests the possibility of gut barrier translocation and increased risk of bloodstream infection^87^.

Aminoglycosides are also combined with β-lactams or vancomycin to treat some gram-positive pathogens, mainly staphylococci but also enterococci^88^. The gene of resistance to vancomycin, *VanHAX*, typical for patients with FN, is usually carried also by *Enterococcus* spp.^89^. It was associated with increased mortality in patients with FN due to the prolonged duration of bacteraemia^90^. Tight correlation of resistance genes, including *msr(C), dfrG, emr(T),* and *erm(B),* can suggest their colocalization within one genome or cooccurrence of bacterial species within the gut. *msr(C)* gene is a chromosomal-encoded ABC-F subfamily protein carried by *E. faecium* that confers resistance to erythromycin and other macrolide and streptogramin B antibiotics. In a study by Mc Gann (2023), a multidrug-resistant strain of *E. faecium* carrying resistance genes *dfrG*, *erm(B)*, and *msr(C)* has been identified^91^. Together with *E. faecium* also, *Lactococcus garviae* has been detected as a multidrug-resistant strain carrying an antimicrobial resistance gene at the plasmid^92^.

For immunocompromised patients, often, hospital-acquired infections are challenging to cure because opportunistic pathogens have accumulated multidrug resistance mechanisms. In our study, the gut resistome of patients with FN has been found to be mainly carried by multidrug-resistant pathobionts such as *E. faecium, Enterococcus faecalis*, *S. aureus*, and *Klebsiella* spp. These pathobionts carried a significant subset of resistance genes and have been suggested to be involved in the patient's treatment and metabolism of trimethoprim / sulfamethoxazole, amikacin, piperacilline/tazobactam, and teicoplanin potentially leading to alterations in the efficiency of the treatment. Resistance to piperacillin / tazobactam was mainly conferred by the genera *K. pneumoniae* and *E. coli*, consistent with other studies findings^93,94^. Bacteraemia caused by gram-positive bacteria was commonly associated with FN, with varying prevalence of gram-negative pathobionts. Prophylactic antibiotics, such as fluoroquinolones and ciprofloxacin, were linked to increased resistance among cultivation-analysed bacteria.

Except for antibiotic treatment, it is crucial also to consider the impact of MTX treatment on the gut barrier and gut microbiome status of patients, as it can contribute to an increased risk of bloodstream infections through bacterial translocation. MTX, a chemotherapy drug also used for the treatment of different types of oncology diagnoses, including acute lymphoblastic leukaemia and non-Hodkgin’s lymphoma, has been found to exhibit toxic effects on multiple organs, including the gastrointestinal tract, bone marrow, heart, kidneys, and liver^95,96^. Additionally, the intestinal toxicity of MTX can lead to mucositis, resulting in symptoms such as nausea, bloating, abdominal pain, and diarrhea^97,98^. The drug triggers inflammatory events that can damage intestinal epithelium and submucosal tissue cells^99^. However, certain bacterial species, such as *B. fragilis* and *R. intestinalis*, can enhance the effects of the drug or reduce its toxicity. In particular, *B. fragilis* has been found to have a protective function against MTX-induced inflammatory reactions^100^ We have found no clear indices regarding the association with FN since two patients with detected *Bacteroides* spp. and developed FN, while one with distinct strains o *B. fragilis* did not. However, more profound insight into strain level could indicate crucial differences in *Bacteroides* spp. population composition. During the surgical intervention, damaged intestinal barrier integrity, bacterial contamination, and the weak immune system of patients, *B. fragilis* can be found in 30–60% of cases of purulent-septic infections. There are two types of *B. fragilis*, either harboring silent resistance gene *cfiA* and with high probability resistance to carbapenems or not. Ank et al. (2015) isolated multidrug-resistant *B.fragilis*^101^ from blood and *B.fragilis* strains detected in the gut microbiome of patients with FN. Strains O17, and O18 harbor th antibiotic resistance genes *cfiA*, *tetQ,* and *nim*^102^ indicating possible activation of carbapenem resistance through insertion element and leading to possible treatment failure^103^.

Additionally*, R. intestinalis* is an obligately anaerobic bacterium capable of using acetate to produce butyrate due to the butyryl-CoA: acetate CoA transferase^104^. Butyrate has anti-inflammatory and metabolic modulatory effects^105,106^ and serves as a source of energy for enterocytes, enhancing the integrity of the intestinal barrier. Cooccurrence of these bacterial genera can improve the treatment outcome. Vitamin supplementation, specifically vitamin C and B2, has also been shown to alleviate the clinical symptoms of MTX-induced mucositis and increase the growth of certain bacterial strains associated with intestinal inflammation. By examining the effects of these vitamins on the in vitro growth of several bacterial strains intrinsically associated with intestinal inflammation, it was found that vitamin supplementation under oxidative conditions increased the growth of *Blautia coccoides* and *R. intestinalis*^107^. Overall, it is essential to consider the potential impact of the microbiome on cancer treatment outcomes and to explore ways to mitigate the harmful effects of chemotherapy drugs.

Nevertheless, there are some limitations of this study that rely in lower number of involved patients, even though representing all the patients with febrile neutropenia hospitalized during the period of three years. Furthermore, not all the patients were able to provide sample before and after HSCT. Although, the parenteral nutrition and diet supplements were standardized, the use of antimicrobials was altered and adjusted to current health status of the patient. Additionally, the gender balance within analysed groups was slightly shifted towards male patients (11 vs. 7). While eight males developed febrile neutropenia, only four females were diagnosed with fever. Nevertheless, our study is the first one that investigates the gut microbiome composition, diversity and resistome and identifies multidrug-resistant bacteria of paediatric oncology patients undergoing haematopoietic stem cell transplantation before and after HSCT treatment.

Our research indicates that one must consider the diverse combinations of resistant or multidrug-resistant bacteria in a patient's gut microbiota. However, it is crucial to recognize the significance of clustering patients based on their resistome, which should be noticed. It is important to note that the gut resistome has the potential to connect the gut microbiome and the FN, therefore, it is a critical factor that must be considered. Continuous monitoring and adaptation of antimicrobial therapy based on local resistance patterns would be critical for effective management. Judicious use of antibiotics would also be essential in mitigating the emergence and spread of antibiotic-resistant bacteria in febrile neutropenic patients^93–96^.

## Conclusion

Hematopoietic stem cell transplantation is lifesaving but still associated with adverse outcomes. We revealed a patient-specific resistome pattern rather than a typical bacterial profile associated with conditioning regimen treatment in patients undergoing HSCT. However, significant decrease of alpha-diversity was identified in the gut microbiome of patients with FN one week after HSCT. Furthermore, there appears to be a correlation between the treatment consequences and a higher prevalence of specific resistance genes *tetQ* and *cfrA* in the gut microbiome one week after HSCT. This finding is noteworthy, as it may affect future treatments and patient care. It is also important to note that a group of nine resistance genes, including *msr(C)*, *dfrG*, *erm(T)*, *VanHAX*, *erm(B)*, *aac(6)-aph(2)*, *aph(3)-III*, *ant(6)-Ia*, and *aac(6)-Ii*, demonstrate a significant correlation and connection to FN. The gut resistome of patients with FN has been found to be mainly carried by multidrug-resistant pathobionts such as *E. faecium*, *E. faecalis*, *S. aureus*, and *Klebsiella* spp. that have been suggested to be involved in the patient's treatment through metabolism of trimethoprim/sulfamethoxazole, amikacin, piperacilline/tazobactam, and teicoplanin. These findings need to be supported with future studies or information on the state of the intestinal barrier linking the gut with inflammation. It is worth mentioning that some gut bacteria can also be involved in cancer drug metabolism. Our results suggest screening for resistance genes in immunocompromised pediatric patients through quantitative analysis using qPCR or targeted screening for ESKAPE bacteria via PCR methods to improve management of multidrug-resistant infections. A personalized approach is necessary for assessing gut microbiome and resistome of patients undergoing HSCT in order to enhance development of effective treatment strategies and mitigate adverse outcomes.

### Supplementary Information

Supplementary Information.

Supplementary Information.

## Data Availability

Raw data from shotgun metagenomic sequencing have been deposited to SRA database under BioProject ID PRJNA1009258 (Supplementary Table 10). The software packages used in this study are open-source and free.

## Abbreviations

HSCT: Hematopoietic stem cell transplantation
FN: Febrile neutropenia
GvHD: Graft versus host disease
ARB: Antibiotic-resistant bacteria
AMR: Antimicrobial resistance
PCT: Procalcitonin
CRP: C-reactive protein
MTX: Methotrexate
SCFA: Short-chain fatty acid

## References

[1] Satlin MJ, Walsh TJ (2017). Multidrug-resistant enterobacteriaceae, pseudomonas aeruginosa, and vancomycin-resistant enterococcus: Three major threats to hematopoietic stem cell transplant recipients. Transpl. Infect. Dis..

[2] Rashidi A, Kaiser T, Graiziger C, Holtan SG, Rehman TU, Weisdorf DJ (2019). Specific gut microbiota changes heralding bloodstream infection and neutropenic fever during intensive chemotherapy. Leukemia.

[3] Rashidi A, Kaiser T, Shields-Cutler R, Graiziger C, Holtan SG, Rehman TU (2019). Dysbiosis patterns during re-induction/salvage versus induction chemotherapy for acute leukemia. Sci. Rep..

[4] Perez P, Patiño J, Estacio M, Pino J, Manzi E, Medina D (2020). Bacteremia in pediatric patients with hematopoietic stem cell transplantation. Rev. Bras. Hematol. Hemoter..

[5] van Hecke O, Wang K, Lee JJ, Roberts NW, Butler CC (2017). Implications of antibiotic resistance for patients’ recovery from common infections in the community: A systematic review and meta-analysis. Clin. Infect. Dis..

[6] Nanayakkara AK, Boucher HW, Fowler VG, Jezek A, Outterson K, Greenberg DE (2021). Antibiotic resistance in the patient with cancer: Escalating challenges and paths forward. CA A Cancer J. Clin..

[7] Hakim H, Dallas R, Wolf J, Tang L, Schultz-Cherry S, Darling V (2018). Gut microbiome composition predicts infection risk during chemotherapy in children with acute lymphoblastic leukemia. Clin. Infect. Dis..

[8] Nearing, J. T., Connors, J., Whitehouse, S., Van Limbergen, J., Macdonald, T., Kulkarni, K. *et al*. Infectious complications are associated with alterations in the gut microbiome in pediatric patients with acute lymphoblastic Leukemia. *Front. Cell Infect. Microbiol*. [cited 2023 Jul 14];9, (2019). Available from: https://www.frontiersin.org/articles/10.3389/fcimb.2019.00028 10.3389/fcimb.2019.00028 PMC6389711 30838178

[9] Poutsiaka DD, Price LL, Ucuzian A, Chan GW, Miller KB, Snydman DR (2007). Blood stream infection after hematopoietic stem cell transplantation is associated with increased mortality. Bone Marrow Transplant..

[10] Henig I, Zuckerman T (2014). Hematopoietic stem cell transplantation-50 years of evolution and future perspectives. Rambam Maimonides Med. J..

[11] Heston, S. M., Young, R. R., Jenkins, K., Martin, P. L., Stokhuyzen, A., Ward, D. V., *et al*. The gut resistome during hematopoietic stem cell transplantation in children [Internet]. medRxiv; 2022 [cited 2023 Jul 14]. p. 2022.07.07.22277185. Available from: https://www.medrxiv.org/content/10.1101/2022.07.07.22277185v1

[12] Lucas AJ, Olin JL, Coleman MD (2018). Management and preventive measures for febrile neutropenia. Pharm. Therapeut..

[13] Castagnola E, Bagnasco F, Mesini A, Agyeman PKA, Ammann RA, Carlesse F (2021). Antibiotic resistant bloodstream infections in pediatric patients receiving chemotherapy or hematopoietic stem cell transplant: Factors associated with development of resistance, intensive care admission and mortality. Antibiotics.

[14] MacDonald, T., Dunn, K. A., MacDonald, J., Langille, M. G. I., Van Limbergen, J. E., Bielawski, J. P., *et al*. The gastrointestinal antibiotic resistome in pediatric leukemia and lymphoma patients. Frontiers in Cellular and Infection Microbiology [Internet]. 2023 [cited 2023 Aug 31];13. 10.3389/fcimb.2023.1102501 10.3389/fcimb.2023.1102501 PMC9998685 36909730

[15] D’Amico F, Soverini M, Zama D, Consolandi C, Severgnini M, Prete A (2019). Gut resistome plasticity in pediatric patients undergoing hematopoietic stem cell transplantation. Sci. Rep..

[16] Masetti, R., Leardini, D., Muratore, E., Fabbrini, M., D’Amico, F., Zama, D. *et al*. Gut microbiota diversity before allogeneic hematopoietic stem cell transplantation as predictor of mortality in children. Blood. blood.2023020026 (2023). 10.1182/blood.2023020026 PMC10651870 37856089

[17] Masetti R, D’Amico F, Zama D, Leardini D, Muratore E, Ussowicz M (2022). Febrile neutropenia duration is associated with the severity of gut microbiota dysbiosis in pediatric allogeneic hematopoietic stem cell transplantation recipients. Cancers.

[18] Schwabkey ZI, Wiesnoski DH, Chang C-C, Tsai W-B, Pham D, Ahmed SS (2022). Diet-derived metabolites and mucus link the gut microbiome to fever after cytotoxic cancer treatment. Sci. Transl. Med..

[19] Ugrayová S, Švec P, Hric I, Šardzíková S, Kubáňová L, Penesová A (2022). Gut microbiome suffers from hematopoietic stem cell transplantation in childhood and its characteristics are positively associated with intra-hospital physical exercise. Biology.

[20] Zajac-Spychala, O., Kampmeier, S., Lehrnbecher, T., Groll, A. H. Infectious complications in paediatric haematopoetic cell transplantation for acute lymphoblastic leukemia: Current status. Frontiers in Pediatrics [Internet]. 2022 [cited 2023 Jul 30];9. Available from: 10.3389/fped.2021.782530 10.3389/fped.2021.782530 PMC8866305 35223707

[21] Stalder T, Press MO, Sullivan S, Liachko I, Top EM (2019). Linking the resistome and plasmidome to the microbiome. ISME J..

[22] Yao Y, Maddamsetti R, Weiss A, Ha Y, Wang T, Wang S (2022). Intra- and interpopulation transposition of mobile genetic elements driven by antibiotic selection. Nat. Ecol. Evol..

[23] Atlas [Internet]. [cited 2023 Jul 31]. Available from: https://atlas-surveillance.com/#/heatmap/resistance

[24] Tumbarello M, Viale P, Viscoli C, Trecarichi EM, Tumietto F, Marchese A (2012). Predictors of mortality in bloodstream infections caused by Klebsiella pneumoniae carbapenemase-producing *K. pneumoniae*: Importance of combination therapy. Clin. Infect. Dis..

[25] Shankar K, Radhakrishnan V, Vijayakumar V, Ramamoorthy J, Ganesan P, Dhanushkodi M (2018). Prevalence of multi-drug resistant organisms in stool of paediatric patients with acute leukaemia and correlation with blood culture positivity: A single institution experience. Pediatr. Blood Cancer.

[26] Averbuch D, Tridello G, Hoek J, Mikulska M, Akan H, Yanez San Segundo L (2017). Antimicrobial resistance in gram-negative rods causing bacteremia in hematopoietic stem cell transplant recipients: intercontinental prospective study of the infectious diseases working party of the european bone marrow transplantation group. Clin. Infect. Dis..

[27] Haeusler GM, Mechinaud F, Daley AJ, Starr M, Shann F, Connell TG (2013). Antibiotic-resistant Gram-negative bacteremia in pediatric oncology patients–risk factors and outcomes. Pediatr. Infect Dis. J..

[28] Bodro M, Gudiol C, Garcia-Vidal C, Tubau F, Contra A, Boix L (2014). Epidemiology, antibiotic therapy and outcomes of bacteremia caused by drug-resistant ESKAPE pathogens in cancer patients. Supp. Care Cancer.

[29] Girmenia C, Rossolini GM, Piciocchi A, Bertaina A, Pisapia G, Pastore D (2015). Infections by carbapenem-resistant Klebsiella pneumoniae in SCT recipients: A nationwide retrospective survey from Italy. Bone Marrow Transplant..

[30] Zajac-Spychala O, Wachowiak J, Gryniewicz-Kwiatkowska O, Gietka A, Dembowska-Baginska B, Semczuk K (2021). Prevalence, epidemiology, etiology, and sensitivity of invasive bacterial infections in pediatric patients undergoing oncological treatment: A multicenter nationwide study. Microbial. Drug Resist..

[31] Van Camp P-J, Haslam DB, Porollo A (2020). Bioinformatics approaches to the understanding of molecular mechanisms in antimicrobial resistance. Int. J. Mol. Sci..

[32] Afgan E, Baker D, Batut B, van den Beek M, Bouvier D, Cech M (2018). The Galaxy platform for accessible, reproducible and collaborative biomedical analyses: 2018 update. Nucleic Acids Res..

[33] Babraham Bioinformatics - FastQC A Quality Control tool for High Throughput Sequence Data [Internet]. [cited 2023 Jul 14]. Available from: https://www.bioinformatics.babraham.ac.uk/projects/fastqc/

[34] Bolger AM, Lohse M, Usadel B (2014). Trimmomatic: A flexible trimmer for Illumina sequence data. Bioinformatics.

[35] Nurk S, Meleshko D, Korobeynikov A, Pevzner PA (2017). metaSPAdes: A new versatile metagenomic assembler. Genome Res..

[36] Lu J, Rincon N, Wood DE, Breitwieser FP, Pockrandt C, Langmead B (2022). Metagenome analysis using the Kraken software suite. Nat. Protoc..

[37] Dabdoub, S. kraken-biom: Enabling interoperative format conversion for Kraken results (Version 1.2)[Software] Available at https://github.com/smdabdoub/kraken-biom. 2016.

[38] R Core Team R. R: A language and environment for statistical computing. 2013;

[39] McMurdie PJ, Holmes S (2013). phyloseq: An R package for reproducible interactive analysis and graphics of microbiome census data. PLOS ONE..

[40] Camacho C, Coulouris G, Avagyan V, Ma N, Papadopoulos J, Bealer K (2009). BLAST+: Architecture and applications. BMC Bioinform..

[41] Bortolaia V, Kaas RS, Ruppe E, Roberts MC, Schwarz S, Cattoir V (2020). ResFinder 4.0 for predictions of phenotypes from genotypes. J. Antimicrob. Chemother..

[42] Zankari E, Allesøe R, Joensen KG, Cavaco LM, Lund O, Aarestrup FM (2017). PointFinder: A novel web tool for WGS-based detection of antimicrobial resistance associated with chromosomal point mutations in bacterial pathogens. J. Antimicrob. Chemother..

[43] Sayers EW, Bolton EE, Brister JR, Canese K, Chan J, Comeau DC (2022). Database resources of the national center for biotechnology information. Nucleic Acids Res..

[44] Segata N, Izard J, Waldron L, Gevers D, Miropolsky L, Garrett WS (2011). Metagenomic biomarker discovery and explanation. Genome Biol..

[45] Ondov BD, Bergman NH, Phillippy AM (2011). Interactive metagenomic visualization in a Web browser. BMC Bioinform..

[46] Metsalu T, Vilo J (2015). ClustVis: A web tool for visualizing clustering of multivariate data using principal component analysis and heatmap. Nucleic Acids Res..

[47] Ottman N, Reunanen J, Meijerink M, Pietilä TE, Kainulainen V, Klievink J (2017). Pili-like proteins of Akkermansia muciniphila modulate host immune responses and gut barrier function. PLOS ONE.

[48] Wilson, I. D., Nicholson, J. K. The modulation of drug efficacy and toxicity by the gut microbiome. In: Kochhar, S., Martin, F.-P., editors. Metabonomics and Gut Microbiota in Nutrition and Disease [Internet]. London: Springer; 2015 [cited 2023 Jun 28]. p. 323–41. Available from: 10.1007/978-1-4471-6539-2_15

[49] Roberts MC (2005). Update on acquired tetracycline resistance genes. FEMS Microbiol. Lett..

[50] Chopra I, Roberts M (2001). Tetracycline antibiotics: Mode of action, applications, molecular biology, and epidemiology of bacterial resistance. Microbiol. Mol. Biol. Rev..

[51] Balletto E, Mikulska M (2015). Bacterial infections in hematopoietic stem cell transplant recipients. Mediterr. J. Hematol. Infect. Dis..

[52] Spinardi JR, Berea R, Orioli PA, Gabriele MM, Navarini A, Marques MT (2017). *Enterococcus spp*. and *S. aureus* colonization in neutropenic febrile children with cancer. Germs.

[53] Hong T, Wang R, Wang X, Yang S, Wang W, Gao Q (2021). Interplay between the intestinal microbiota and acute graft-versus-host disease: Experimental evidence and clinical significance. Front. Immunol..

[54] Loof TG, Sohail A, Bahgat MM, Tallam A, Arshad H, Akmatov MK (2018). Early lymphocyte loss and increased granulocyte/lymphocyte ratio predict systemic spread of streptococcus pyogenes in a mouse model of acute skin infection. Front. Cell Infect. Microbiol..

[55] Gudiol C, Bodro M, Simonetti A, Tubau F, González-Barca E, Cisnal M (2013). Changing aetiology, clinical features, antimicrobial resistance, and outcomes of bloodstream infection in neutropenic cancer patients. Clin. Microbiol. Infect..

[56] Skaarud KJ, Hov JR, Hansen SH, Kummen M, Valeur J, Seljeflot I (2021). Mortality and microbial diversity after allogeneic hematopoietic stem cell transplantation: Secondary analysis of a randomized nutritional intervention trial. Sci. Rep..

[57] Taur Y, Jenq RR, Perales M-A, Littmann ER, Morjaria S, Ling L (2014). The effects of intestinal tract bacterial diversity on mortality following allogeneic hematopoietic stem cell transplantation. Blood.

[58] Malard F, Gasc C, Plantamura E, Doré J (2018). High gastrointestinal microbial diversity and clinical outcome in graft-versus-host disease patients. Bone Marrow Transplant..

[59] Rattanathammethee T, Piriyakhuntorn P, Hantrakool S, Chai-Adisaksopha C, Rattarittamrong E, Tantiworawit A (2020). Gut microbiota profiles of treatment-naïve adult acute myeloid Leukemia patientswith neutropenic fever during intensive chemotherapy. Blood.

[60] Bilinski J, Robak K, Peric Z, Marchel H, Karakulska-Prystupiuk E, Halaburda K (2016). Impact of gut colonization by antibiotic-resistant bacteria on the outcomes of allogeneic hematopoietic stem cell transplantation: A retrospective, single-center study. Biol. Blood Marrow Transplant..

[61] Punnapuzha, S., Edemobi, P. K., Elmoheen, A. Febrile neutropenia. StatPearls [Internet]. Treasure Island (FL): StatPearls Publishing; 2023 [cited 2023 Jun 28]. Available from: http://www.ncbi.nlm.nih.gov/books/NBK541102/31082146

[62] Holler E, Butzhammer P, Schmid K, Hundsrucker C, Koestler J, Peter K (2014). Metagenomic analysis of the stool microbiome in patients receiving allogeneic stem cell transplantation: Loss of diversity is associated with use of systemic antibiotics and more pronounced in gastrointestinal graft-versus-host disease. Biol. Blood Marrow Transplant..

[63] Taur Y, Xavier JB, Lipuma L, Ubeda C, Goldberg J, Gobourne A (2012). Intestinal domination and the risk of bacteremia in patients undergoing allogeneic hematopoietic stem cell transplantation. Clin. Infect. Dis..

[64] Greco R, Nitti R, Mancini N, Pasciuta R, Lorentino F, Lupo-Stanghellini MT (2021). Microbiome markers are early predictors of acute GVHD in allogeneic hematopoietic stem cell transplant recipients. Blood.

[65] Ilett EE, Jørgensen M, Noguera-Julian M, Nørgaard JC, Daugaard G, Helleberg M (2020). Associations of the gut microbiome and clinical factors with acute GVHD in allogeneic HSCT recipients. Blood Adv..

[66] Smith AB, Jenior ML, Keenan O, Hart JL, Specker J, Abbas A (2022). Enterococci enhance clostridioides difficile pathogenesis. Nature..

[67] Morrison DJ, Preston T (2016). Formation of short chain fatty acids by the gut microbiota and their impact on human metabolism. Gut. Microbes..

[68] Montassier E, Al-Ghalith GA, Ward T, Corvec S, Gastinne T, Potel G (2016). Pretreatment gut microbiome predicts chemotherapy-related bloodstream infection. Genome Med..

[69] Romick-Rosendale LE, Haslam DB, Lane A, Denson L, Lake K, Wilkey A (2018). Antibiotic exposure and reduced short chain fatty acid production after hematopoietic stem cell transplant. Biol. Blood Marrow Transplant..

[70] Derrien M, Collado MC, Ben-Amor K, Salminen S, de Vos WM (2008). The mucin degrader *Akkermansia muciniphila* is an abundant resident of the human intestinal tract. Appl. Environ. Microbiol..

[71] Earley H, Lennon G, Balfe Á, Coffey JC, Winter DC, O’Connell PR (2019). The abundance of *Akkermansia muciniphila* and its relationship with sulphated colonic mucins in health and ulcerative colitis. Sci. Rep..

[72] Rashidi A, Ebadi M, Rehman TU, Elhusseini H, Nalluri H, Kaiser T (2021). Altered microbiota-host metabolic cross talk preceding neutropenic fever in patients with acute leukemia. Blood Adv..

[73] McInnes RS, McCallum GE, Lamberte LE, van Schaik W (2020). Horizontal transfer of antibiotic resistance genes in the human gut microbiome. Curr. Opin. Microbiol..

[74] Sóki J, Wybo I, Hajdú E, Toprak NU, Jeverica S, Stingu C-S (2020). A Europe-wide assessment of antibiotic resistance rates in Bacteroides and Parabacteroides isolates from intestinal microbiota of healthy subjects. Anaerobe.

[75] Waters JL, Salyers AA (2013). Regulation of CTnDOT conjugative transfer is a complex and highly coordinated series of events. mBio.

[76] Costa Y, Galimand M, Leclercq R, Duval J, Courvalin P (1993). Characterization of the chromosomal aac(6’)-Ii gene specific for *Enterococcus faecium*. Antimicrob. Agents Chemother..

[77] Rice LB (2008). Federal funding for the study of antimicrobial resistance in nosocomial pathogens: No ESKAPE. J. Infect. Dis..

[78] Ramirez MS, Tolmasky ME (2010). Aminoglycoside modifying enzymes. Drug. Resist. Updat..

[79] Shaul P, Green KD, Rutenberg R, Kramer M, Berkov-Zrihen Y, Breiner-Goldstein E (2011). Assessment of 6’- and 6’’’-N-acylation of aminoglycosides as a strategy to overcome bacterial resistance. Org. Biomol. Chem..

[80] Herzog IM, Green KD, Berkov-Zrihen Y, Feldman M, Vidavski RR, Eldar-Boock A (2012). 6”-Thioether tobramycin analogues: Towards selective targeting of bacterial membranes. Angew. Chem. Int. Ed. Engl..

[81] Darakhshandeh A, Fathi E, Haji Gholami A, Ashrafi F, Mehrzad V, Nasri E (2023). Bacterial spectrum and antimicrobial resistance pattern in cancer patients with febrile neutropenia. Int. J. Biochem. Mol. Biol..

[82] Chow JW (2000). Aminoglycoside resistance in enterococci. Clin. Infect. Dis..

[83] Shaw KJ, Rather PN, Hare RS, Miller GH (1993). Molecular genetics of aminoglycoside resistance genes and familial relationships of the aminoglycoside-modifying enzymes. Microbiol. Rev..

[84] Wang M, Tran JH, Jacoby GA, Zhang Y, Wang F, Hooper DC (2003). Plasmid-mediated quinolone resistance in clinical isolates of *Escherichia coli* from Shanghai, China. Antimicrob. Agents Chemother..

[85] Kim ES, Jeong J-Y, Jun J-B, Choi S-H, Lee S-O, Kim M-N (2009). Prevalence of aac(6′)-Ib-cr encoding a ciprofloxacin-modifying enzyme among enterobacteriaceae blood isolates in Korea. Antimicrob. Agents Chemother..

[86] Shen P, Jiang Y, Zhou Z, Zhang J, Yu Y, Li L (2008). Complete nucleotide sequence of pKP96, a 67 850 bp multiresistance plasmid encoding qnrA1, aac(6′)-Ib-cr and blaCTX-M-24 from Klebsiella pneumoniae. J. Antimicrob. Chemother..

[87] Choi H, Ahn H, Lee R, Cho S-Y, Lee D-G (2022). Bloodstream infections in patients with hematologic diseases: Causative organisms and factors associated with resistance. Infect. Chemother..

[88] Yao, J. D. C., Moellering, Jr. R. C. Antibacterial agents. Manual of Clinical Microbiology [Internet]. John Wiley & Sons, Ltd; 2011 [cited 2023 Jul 31]. p. 1041–81. 10.1128/9781555816728.ch65

[89] Kohler P, Eshaghi A, Kim HC, Plevneshi A, Green K, Willey BM (2018). Prevalence of vancomycin-variable *Enterococcus faecium* (VVE) among vanA-positive sterile site isolates and patient factors associated with VVE bacteremia. PLOS ONE.

[90] DiazGranados CA, Jernigan JA (2005). Impact of vancomycin resistance on mortality among patients with neutropenia and enterococcal bloodstream infection. J. Infect. Dis..

[91] Gann, P. T. M., Lebreton, F., Jones, B. T., Dao, H. D., Martin. M. J., Nelson, M. J., *et al*. Six extensively drug-resistant bacteria in an injured soldier, Ukraine - Volume 29, Number 8—August 2023 - Emerging Infectious Diseases journal - CDC. [cited 2023 Jul 31]; Available from: https://wwwnc.cdc.gov/eid/article/29/8/23-0567_article 10.3201/eid2908.230567 PMC10370857 37406356

[92] Cai J, Chen J, Schwarz S, Wang Y, Zhang R (2021). Detection of the plasmid-borne oxazolidinone/phenicol resistance gene optrA in Lactococcus garvieae isolated from faecal samples. Clin. Microbiol. Infect..

[93] Joudeh N, Sawafta E, Abu Taha A, Hamed Allah M, Amer R, Odeh RY (2023). Epidemiology and source of infection in cancer patients with febrile neutropenia: An experience from a developing country. BMC Infect. Dis..

[94] Edwards, T., Heinz, E., Aartsen J. van, Howard, A., Roberts, P., Corless, C. *et al*. Piperacillin/tazobactam resistant, cephalosporin susceptible Escherichia coli bloodstream infections driven by multiple resistance mechanisms across diverse sequence types [Internet]. bioRxiv; 2020 [cited 2023 Jun 28]. p. 2020.09.18.302992. Available from: 10.1101/2020.09.18.302992v2

[95] Perez-Verdia A, Angulo F, Hardwicke FL, Nugent KM (2005). Acute cardiac toxicity associated with high-dose intravenous methotrexate therapy: Case report and review of the literature. Pharmacotherapy.

[96] Widemann BC, Balis FM, Kempf-Bielack B, Bielack S, Pratt CB, Ferrari S (2004). High-dose methotrexate-induced nephrotoxicity in patients with osteosarcoma. Cancer.

[97] Avritscher EBC, Cooksley CD, Elting LS (2004). Scope and epidemiology of cancer therapy-induced oral and gastrointestinal mucositis. Semin. Oncol. Nurs..

[98] Ben-Lulu S, Pollak Y, Mogilner J, Bejar JG, Coran A, Sukhotnik I (2012). Dietary transforming growth factor-beta 2 (TGF-β2) supplementation reduces methotrexate-induced intestinal mucosal injury in a rat. PLoS One.

[99] Sonis ST (2004). The pathobiology of mucositis. Nat. Rev. Cancer.

[100] Zhou B, Xia X, Wang P, Chen S, Yu C, Huang R (2018). Induction and amelioration of methotrexate-induced gastrointestinal toxicity are related to immune response and gut microbiota. EBioMedicine.

[101] Ank N, Sydenham TV, Iversen LH, Justesen US, Wang M (2015). Characterisation of a multidrug-resistant Bacteroides fragilis isolate recovered from blood of a patient in Denmark using whole-genome sequencing. Int. J. Antimicrob. Agents.

[102] Kozhakhmetova S, Zholdybayeva E, Tarlykov P, Atavliyeva S, Syzdykov T, Daniyarov A (2021). Determinants of resistance in Bacteroides fragilis strain BFR_KZ01 isolated from a patient with peritonitis in Kazakhstan. J. Global Antimicrob. Resist..

[103] Edwards R, Read PN (2000). Expression of the carbapenemase gene (cfiA) in Bacteroides fragilis. J. Antimicrob. Chemother..

[104] Pryde SE, Duncan SH, Hold GL, Stewart CS, Flint HJ (2002). The microbiology of butyrate formation in the human colon. FEMS Microbiol. Lett..

[105] Canfora EE, Jocken JW, Blaak EE (2015). Short-chain fatty acids in control of body weight and insulin sensitivity. Nat. Rev. Endocrinol..

[106] McNabney SM, Henagan TM (2017). Short chain fatty acids in the colon and peripheral tissues: A focus on butyrate, colon cancer, obesity and insulin resistance. Nutrients.

[107] da Silva Ferreira AR, Wardill HR, Havinga R, Tissing WJE, Harmsen HJM (2020). Prophylactic treatment with vitamins C and B2 for methotrexate-induced gastrointestinal mucositis. Biomolecules.

