# Supplementary material for: Gut diversity and the resistome as biomarkers of febrile neutropenia outcome in paediatric oncology patients undergoing hematopoietic stem cell transplantation
Source: Sci Rep. 2024 Mar 6;14:5504. doi: 10.1038/s41598-024-56242-8 (PMC10918076; doi:10.1038/s41598-024-56242-8)
Supplement: Supplementary file 2 — Supplementary Information. [file 41598_2024_56242_MOESM2_ESM.pdf]

Supplementary data

Supplementary data related to this study include tables. Supplementary Table 1: Metadata of the samples; Supplementary Table 2: The antimicrobial treatment of the individual patients, Supplementary Table 3: Shotgun metagenomic sequencing statistics; Supplementary Table 4: Relative abundance of bacterial phyla; Supplementary Table 5: Relative abundance of bacterial families; Supplementary Table 6: The presence of the resistance genes in the individual samples; Supplementary Table 7: List of antibiotic resistant genes and their annotation; Supplementary Table 8: Significantly different bacterial genera in the samples of patients with febrile neutropenia before/after febrile neutropenia outcome; Supplementary Table 9: Selected immune cells status and inflammation biomarkers; Supplementary Table 10: BioSamples data information.
